# Chromosome doubling mediates superior drought tolerance in *Lycium ruthenicum* via abscisic acid signaling

**DOI:** 10.1038/s41438-020-0260-1

**Published:** 2020-04-01

**Authors:** Shupei Rao, Yuru Tian, Xinli Xia, Yue Li, Jinhuan Chen

**Affiliations:** 1 Beijing Advanced Innovation Center for Tree Breeding by Molecular Design, Beijing Forestry University, 100083 Beijing, China; 2 College of Biological Sciences and Technology, Beijing Forestry University, 100083 Beijing, China; 3 National Engineering Laboratory for Tree Breeding, Beijing Forestry University, 100083 Beijing, China

**Keywords:** Abiotic, Polyploidy in plants

## Abstract

Plants are continuously affected by unfavorable external stimuli, which influences their productivity and growth. Differences in gene composition and expression patterns lead homologous polyploid plants to exhibit different physiological phenomena, among which enhanced environmental adaptability is a powerful phenotype conferred by polyploidization. The mechanisms underlying the differences in stress tolerance between diploids and autotetraploids at the molecular level remain unclear. In this research, a full-length transcription profile obtained via the single-molecule real-time (SMRT) sequencing of high-quality single RNA molecules for use as background was combined with next-generation transcriptome and proteome technologies to probe the variation in the molecular mechanisms of autotetraploids. Tetraploids exhibited an increase in ABA content of 78.4% under natural conditions and a superior stress-resistance phenotype under severe drought stress compared with diploids. The substantial differences in the transcriptome profiles observed between diploids and autotetraploids under normal growth conditions were mainly related to ABA biosynthesis and signal transduction pathways, and *9-cis-epoxycarotenoid dioxygenase 1* (*NCED1*) and *NCED2*, which encode key synthetic enzymes, were significantly upregulated. The increased expression of the *ABRE-binding factor 5-like* (*ABF5-like*) gene was a pivotal factor in promoting the activation of the ABA signaling pathway and downstream target genes. In addition, ABA strongly induced the expression of osmotic proteins to increase the stress tolerance of the plants at the translational level. We consider the intrinsic mechanisms by which ABA affects drought resistance in tetraploids and diploids to understand the physiological and molecular mechanisms that enhance abiotic stress tolerance in polyploid plants.

## Introduction

Plants growing under field conditions are continuously affected by unfavorable external stimuli, including abiotic and biotic stresses, which influence their growth and productivity. Abiotic stresses, especially drought, salinity, and temperature, are critical environmental elements that affect the geographical distribution of plants^1^. Commercial forests with poor drought-stress resistance consume excessive resources and greatly increase environmental pressure, so the amelioration of plant stress resistance is crucial for environmental sustainability^2^. Polyploidy refers to the presence of more than two haploid genomes in a single cell nucleus of an organism and has marked effects on plant evolution and species diversity^3^. With genome doubling, the structure and function of the genome are altered, and novel traits and changes in existing physiological processes arise over time^4^. Increased adaptability to environmental variability is a powerful ecological phenotype conferred by polyploidization^5^. Polyploidization in poplar^6^, black locust^7^, and other plant species has contributed to a marked improvement in stress resistance. Allario et al.^8^ used gene expression analyses to examine the differences in drought responses between diploid and autotetraploid clones of *Citrus limonia*. However, the mechanism underlying the increased stress resistance of polyploids remains unclear.

*Lycium ruthenicum* is a perennial spiny shrub that belongs to the Solanaceae family. This family includes numerous species that are commonly used to study plant growth patterns and is considered a model for connecting genomics with biodiversity^9,10^. *L. ruthenicum* is a wild commercial resource native to the saline–alkali arid region of Northwest China and possesses extremely high abiotic stress tolerance properties. This plant is useful for improving soil and water conservation and is a unique desert-specialist species^11^. *L. ruthenicum*, which is rich in natural anthocyanins that perform free radical scavenging and antioxidant functions, plays a vital role not only in the ecosystem but also as a healthy food and medicinal plant^12^. The polyploidization of *L. ruthenicum* may further strengthen its resistance to stresses and is of profound importance for the cultivation of novel germplasm resources in saline–alkali soil and rainless areas. In this regard, *L. ruthenicum* is an ideal experimental material that can be used to study the mechanisms of abiotic stress tolerance that are enhanced by chromosome doubling.

High-throughput omics techniques are widely used to study the interactions of plants with other factors^13^. High-throughput transcriptome sequencing is an important means of understanding phenotype expression and function and is the basis of research on gene function and structure. This method can be used to explore the transcription and regulation of genes in cells at the molecular level^14^. Many non-model plants lack reference genomic information, and second-generation sequencing reads are too short for this purpose, which greatly impedes the capacity to estimate genome-wide transcript abundance. For such plants, full-length transcriptome sequencing of high-quality single RNA molecules is desirable^15^. The PacBio platform based on single-molecule real-time (SMRT) sequencing technology has gained popularity for performing full-length sequencing^16^. Some scholars have conducted intensive research on the metabolic pathways and stress resistance of floriculture species^17^, vegetables^18^, and cash crops^19^ using SMRT sequencing technology. Recently, proteomics has become a complementary technology to transcriptomics with the rapid development of mass spectrometry and quantitative methods^20^. However, research combining full-length transcriptome data with proteomics to reveal ploidy-related molecular mechanisms in *L. ruthenicum* has not been reported previously.

In our previous studies, tetraploid plants of *L. ruthenicum* were efficiently obtained by treating their leaves with colchicine in vitro, and the highest frequency of polyploidy induction was 31.4%^21^. To improve abiotic stress resistance in polyploid perennial woody plants, we utilized autotetraploid *L. ruthenicum* germplasm and evaluated the performance of diploid and tetraploid plants under drought stress. The study was aimed at the three following targets: first, obtaining the full-length transcriptional and protein profiles of *L. ruthenicum*; second, evaluating the differences in drought-resistance performance between diploid and autotetraploid *L. ruthenicum*; and finally, revealing the drought-resistance mechanism of autotetraploids. These results lay a foundation for understanding the mechanism of improved drought resistance in polyploidized *L. ruthenicum*. The results are critical for research on the development of novel economic crops with increased stress resistance.

## Materials and methods

### Plant materials and ploidy identification

Autotetraploid individuals of *L. ruthenicum* were derived from diploid plants treated with colchicine following the method used in our previous study^21^. The first step of the method was to collect fully expanded leaves from a single plant line for shoot regeneration. The explants were cut into 0.5 × 0.5 cm square pieces and precultured in MS medium for 10 days. When calli appeared at the edges of the leaves, they were transferred to liquid medium that contained 1% (v/v) dimethyl sulfoxide (Biodee, Beijing, China) and 100 mg/L colchicine (Biodee, Beijing, China) for 48 h in the dark. After the colchicine induction treatment, the explants were washed three times with sterile water and transferred to MS medium to obtain adventitious buds.

The putative sterile tetraploid and control diploid plants were sliced into 1 cm petioles with at least one axillary bud and transferred to MS medium containing 0.49 μM indole-3-butyric acid (Biodee, Beijing, China) at pH 6.0. The plants with more than eight leaves were subsequently transplanted into soil, containing a mixture of turfy soil and vermiculite, and grown in a greenhouse at a temperature of 24 ± 1 °C under a 16 h photoperiod with a 3 klx intensity of cool white fluorescent light. The plants were cultured in the greenhouse for 1 month under the same environmental conditions and subjected to ploidy detection by flow cytometry (Partec-PAS, Münster, Germany). We performed the analysis three times per plant. The leaves were cut into square fragments in a plastic petri dish, and 1 mL of lysate (pH 7.0) was added. After filtering into a flow tube, 200 µL of 4’,6-diamidino-2-phenylindole (DAPI, 10 µg/mL) fluorescence staining solution (Biodee, Beijing, China) was added for 1–2 min in the dark, and polyploidy was subsequently detected by using a Cyflow^®^ Ploidy Analyzer (Partec, Hesse-Darmstadt, Germany). The standard peak of the diploid control was adjusted to channel 50.

### Drought-stress treatment

To investigate whether the drought-resistance phenotype of tetraploids is better than that of diploids, *L. ruthenicum* plants with different ploidies that were grown under the same environmental conditions and were of uniform height were subjected to drought treatment. Drought treatment was performed under soil culture conditions. For the drought treatment, 2-month-old diploid and tetraploid tissue-cultured seedlings were transplanted to mixed soil (turfy soil:vermiculite, 3:1, v/v) and cultured in an artificial climate chamber until their growth was stable. According to the drought gradient standard, drought treatment is considered to be initiated when the soil moisture content is ~15% or the soil relative humidity is ~60% of the water-holding capacity^22^. Drought treatment was conducted by withholding water for 15 days to determine the drought-resistance phenotype.

### Chlorophyll and hydrogen peroxide determination

To determine whether the tetraploid plants presented higher resistance than the diploid plants after drought-stress treatment, we detected the chlorophyll content and the peroxide index to determine the performance of these plants at the physiological level. Chlorophyll extraction was carried out in 0.1 g of leaves from plants subjected to drought-stress for 0, 8, or 12 days. Chlorophyll was extracted by using 95% ethanol in the dark, and the extract was filtered into a 10-ml test tube. The absorbance of the solution was measured at 665, 649, and 470 nm. The hydrogen peroxide content was determined colorimetrically based on peroxidase activity on the 12th day of drought treatment using a diaminobenzidine (DAB) chromogenic kit (Solarbio, Beijing, China) in accordance with the manufacturer’s instructions. The brown precipitate produced in the leaf tissue was observed microscopically using a ×20 objective lens (Olympus CX23, Tokyo, Japan).

### Determination of abscisic acid content in a normal environment by ultra-performance liquid chromatography–mass spectrometry (UPLC-MS/MS)

Approximately 50 mg of powder from the same tissues used for sequencing was obtained per tube at low temperature and immediately weighed with a 1/10,000 balance. Approximately 0.5 mL of the extract solution (isopropanol:H_2_O:HCl = 2:1:0.002, v/v/v) was mixed with the powder in a centrifuge tube, and 10 μL of 1 ng μL^−1^ D6 abscisic acid (ABA) was added as an internal standard. The subsequent UPLC-MS/MS extraction procedure followed the method with slight modifications^23^. Approximately 50 µL of the extracted sample solution was injected into a reverse-phase C18 Gemini HPLC column for analysis. The ABA content was determined using ultra-performance liquid chromatography–electrospray ionization triple quadrupole mass spectrometry (UPLC-ESI-MS/MS) (Agilent 5500, Santa Clara, CA, USA). The areas of the peaks in the chromatogram were quantified using MassHunter software (Agilent, Santa Clara, CA, USA).

### PacBio library preparation, sequencing, and annotation of SMRT reads

Approximately 0.2 g of healthy leaf tissue from the plants of each ploidy level was sampled for sequencing. The SMARTer™ PCR cDNA Synthesis Kit (Takara Bio USA, Mountain View, CA, USA) was used to synthesize full-length cDNA from total RNA extracts, which was amplified by high-throughput PCR. The ends of the amplified full-length cDNA were repaired. SMRT dumbbell-type adapters were ligated and subjected to exonuclease digestion to generate a sequenceable library. After the quality test, a SMRT^®^ Cell was used to perform full-length transcriptome sequencing without the interruption of the RNA fragments to obtain full-length cDNA.

The raw polymerase read fragment sequences with a length of <50 bp or sequence accuracy of <0.90 were filtered out. After trimming the junction adapters from the remaining sequences, the subreads with a length of >50 bp were screened as clean data. The reads corresponding to inserts in the circular consensus sequence (CCS) reads were removed using the following parameters: full passes ≥1 and sequence accuracy >0.90. Similar sequences among the full-length non-chimeric sequences were clustered using Iso-Seq^®^ with SMRT^®^ Link software (Pacific Biosciences of California, Inc., DE, USA). A consensus isoform was selected for each cluster. Redundant sequences were removed using CD-HIT, resulting in a nonredundant transcript sequence^24^.

The nonredundant transcript sequences were mapped to seven public databases: nr (NCBI nonredundant protein sequences; http://www.ncbi.nlm.nih.gov/RefSeq/)^25^, Swiss-Prot (http://www.UniProt.org/)^26^, GO (Gene Ontology; http://www.geneontology.org/)^27^, COG (Clusters of orthologous groups; http://www. ncbi.nlm. nih.gov/COG)^28^, KOG (Clusters of euKaryotic Orthologous Groups; http://www.ncbi.nlm.nih.gov/KOG)^29^, Pfam (Protein family; http://pfam.xfam.org)^30^, and KEGG (Kyoto Encyclopedia of Genes and Genomes; http://www.genome.jp/kegg)^31^, using BLAST software (E-value ≤ 10^−5^, https://blast.ncbi.nlm.nih.gov/Blast.cgi) to obtain annotation information for the transcripts^32^.

### Bioinformatic characterization with Illumina RNA-Seq and iTRAQ proteomics technology

The mRNA was isolated from the total RNA extracted from each of the six samples using oligo dT primers. Six libraries were generated and purified using the NEBNext^®^ Ultra™ RNA Library Prep Kit for Illumina^®^ (New England Biolabs Inc., Ipswich, MA, USA) and AMPure XP Beads (Beckman Coulter, Inc., Indianapolis, IN, USA) with fragmented mRNA as the template, following the manufacturer’s recommendations. The concentration, integrity, and quantification of the libraries were determined using a Qubit™ Fluorometer (Thermo Fisher Scientific, Waltham, MA, USA), the KAPA Library Quantification Kit (KAPA Biosystems, Wilmington, MA, USA), and a Qsep100 DNA Analyzer (KAPA Biosystems, Wilmington, MA, USA), respectively. The denatured libraries were subjected to high-throughput parallel sequencing of both ends of the sequences using the Illumina HiSeq X™ Ten System sequencing platform (New England Biolabs Inc., Ipswich, MA, USA). The clean data were separated using Cutadapt (https://cutadapt.readthedocs.io/en/stable/), and the quality threshold was set to Q30, resulting in the removal of sequencing adapters and the primer sequence from the raw data to filter out low-quality data^33^. The clean data were aligned to the nonredundant transcripts using STAR (https://github.com/alexdobin/STAR). Transcript levels were quantified using RSEM software (https://github.com/deweylab/RSEM), and the lengths of the transcripts in the samples were normalized to fragments per kilobase of exon per million reads mapped (FPKM) values^34^.

Four separate biological experiments were performed to quantify protein expression in the samples using an organic solvent extraction method and determine the concentration of the protein obtained using the bicinchoninic acid protein assay^35^. Protein (10 μg) was separated via 12% SDS-PAGE and stained with Coomassie brilliant blue in accordance with the method of Candiano et al.^36^. Trypsin was subjected to enzymatic hydrolysis and lyophilization, and 40 μL of the lyophilized sample in TEAB buffer was labeled with TMT reagent. Mass spectral peaks detected by HPLC-MS/MS (Agilent 1100 HPLC, Santa Clara, CA, USA) were analyzed using Proteome Discoverer 2.2 software (Thermo Fisher Scientific, Waltham, MA, USA). The results were subjected to BLAST searches against the full-length transcriptome database acquired by sequencing.

### Analysis of differentially expressed genes and proteins based on the full-length transcriptome

Based on the FPKM values, differential expression at the transcript level was analyzed to determine differentially expressed genes (DEGs) among the samples using DESeq^37^. The criteria were total mapping reads ≥10, a log_2_ fold change (FC) ≥ 1 or ≤ −1, and a *P*-value < 0.05 after false discovery rate correction. The raw protein data were selected for authentic proteins according to the criteria of a Score Sequest HT > 0 and unique peptides ≥ 1. Differentially expressed proteins (DEPs) were screened from among the authentic proteins by comparing those with a FC > 1.2 or FC < 5/6, and differences were considered significant at a *P*-value < 0.05. Functional annotation using the GO and KEGG databases was performed on the differentially expressed transcripts and proteins. The GO and KEGG databases were used to further interpret the functions of the proteins. GO enrichment analysis was performed for the differentially expressed transcripts among the samples. The enriched terms were used to generate a directed acyclic graph using the R package ‘topGO’. Hypergeometric testing was applied to identify pathways that were significantly enriched among the differentially expressed transcripts. The OmicsBean data integration analysis cloud platform was used to perform further GO and KEGG functional annotation and enrichment analysis of the differentially expressed proteins. Transcription factors (TFs) are proteins that bind to a specific nucleotide sequence upstream of a gene that regulates the binding of RNA polymerase to the DNA template to control gene transcription. The TFs among the differentially expressed genes were predicted using iTAK software^38^.

### Validation of DEGs by quantitative real-time PCR

Total RNA was extracted using a plant polysaccharide polyphenol RNA kit (TIANGEN Biotech Co., Ltd, Beijing, China). For each sample, 1 ng of RNA was used as the template for reverse transcription to obtain the same concentration of cDNA using a reverse transcription kit (Aidlab Biotechnologies Co., Ltd, Beijing, China). The primer sequences used are shown in Supplementary Table S1. Reactions were performed in a 20-µL volume using the 2× SYBR^®^ Green qPCR Mix Kit (Aidlab Biotechnologies Co., Ltd), 1 µL of cDNA and the designed primers. Reactions were performed in an ABI PRISM 7500 real-time PCR system (Applied Biosystems, Foster City, CA, USA) in three steps. The real-time PCR data were analyzed using the 2^−∆∆Ct^ method^39^.

## Results

### Ploidy verification and phenotypic variation in drought-treated *L. ruthenicum*

**Fig. 1:**
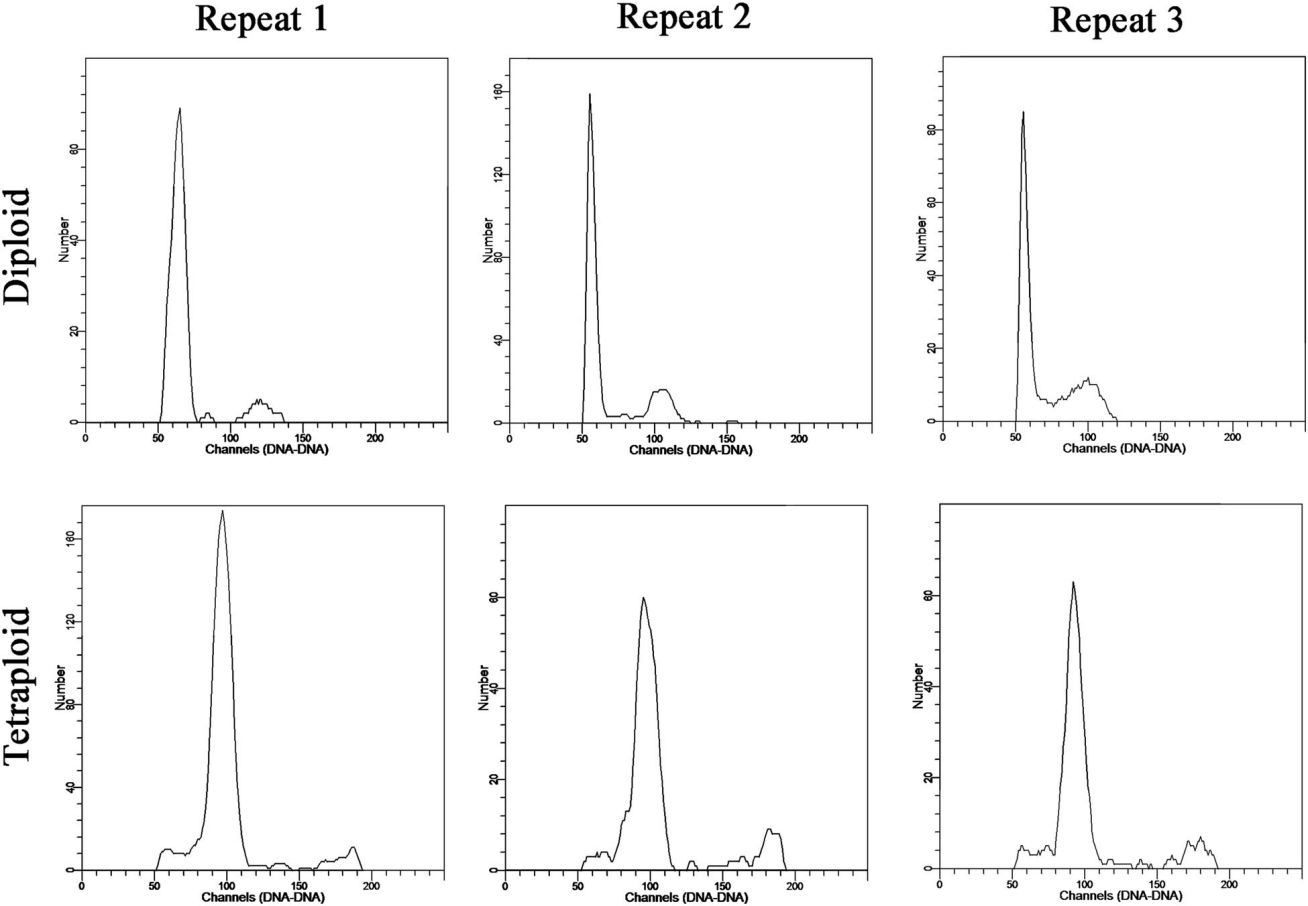
Target plants for the detection of ploidy by flow cytometry. The upper part of the flow cytometry diagram shows the results for three diploid samples, and the bottom shows the results for tetraploids. The *X* and *Y* axes represent the ploidy and the number of cells, respectively

**Fig. 2:**
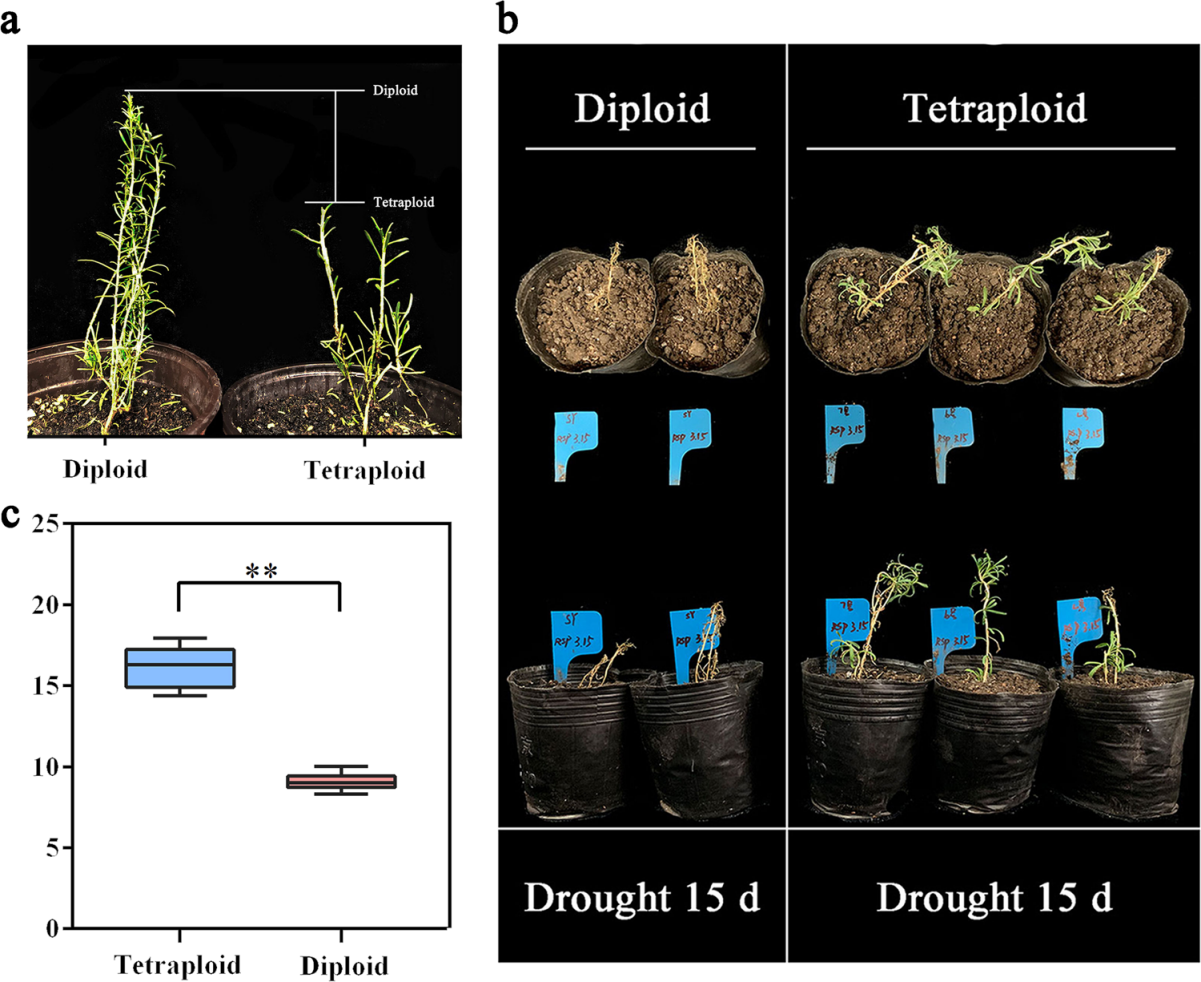
Phenotypic status under stress and ABA content determination in *L. ruthenicum* of different ploidies. **a** Phenotypes in *L. ruthenicum* of different ploidies grown in the greenhouse for 1 month. **b** Phenotypic status of *L. ruthenicum* seedlings under salt and drought stress. **c** Box plot of ABA contents. For each sample three replicates were performed. The two images on the left present the growth of diploids and tetraploids at 148 h under 350 mM salt treatment, and the two figures on the right show the growth of diploids and tetraploids after 15 days of drought. Duncan’s test was applied to determine significant differences between the different ploidies. Two asterisks on a column indicate a significant difference at *p* < 0.01

Autotetraploid individuals of *L. ruthenicum* were obtained by colchicine treatment of the apical leaves of diploid plants following the method used in previous studies^21^. As shown by the histogram of the flow cytometry analysis results, the DNA content of the cells of the colchicine-treated plants was doubled compared with that of the diploid plants (Fig. 1). In terms of phenotype, *L. ruthenicum* plants that differed in ploidy showed conspicuous growth disparities as a result of chromosome doubling, and the growth of the tetraploids was slower than that of the diploids (Fig. 2a).

Drought is a comparatively severe form of abiotic stress. The diploid and autotetraploid plants were subjected to drought stress, and their phenotypic responses were determined. Under a reduction in the soil water content to less than 15% for 15 days, the diploid plants were unable to withstand the damage from dehydration, and all plants in this group died (Fig. 2b). The leaves of the diploid plants curled, and the stems began to shrink before death. In contrast, the tetraploid plants were able to grow normally, and the leaves remained green and turgid. Thus, the tetraploid plants exhibited stronger drought resistance than the diploid plants.

**Fig. 3:**
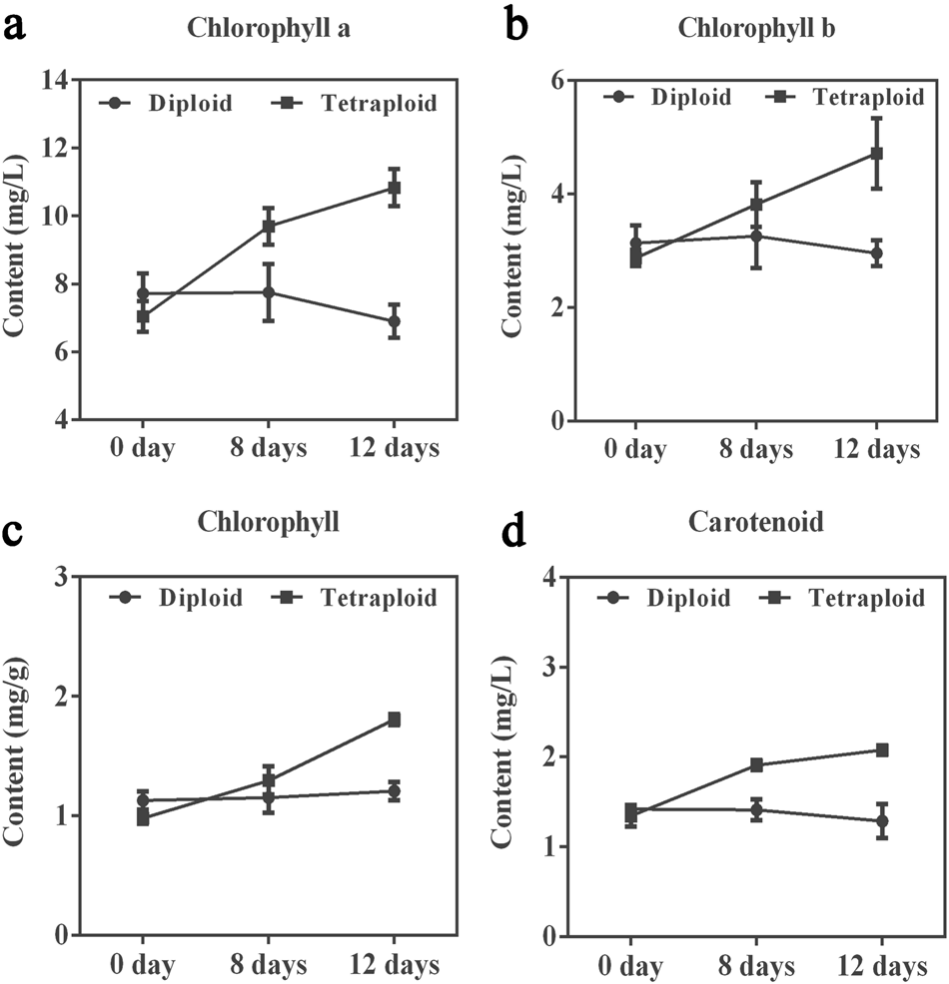
Chlorophyll a, b, total chlorophyll and carotenoid contents after 0, 8, and 12 days of drought. **a** Chlorophyll a after 0, 8, and 12 days of drought. **b** Chlorophyll b after 0, 8, and 12 days of drought. **c** Total chlorophyll content after 0, 8, and 12 days of drought. **d** Carotenoids after 0, 8, and 12 days of drought

**Fig. 4:**
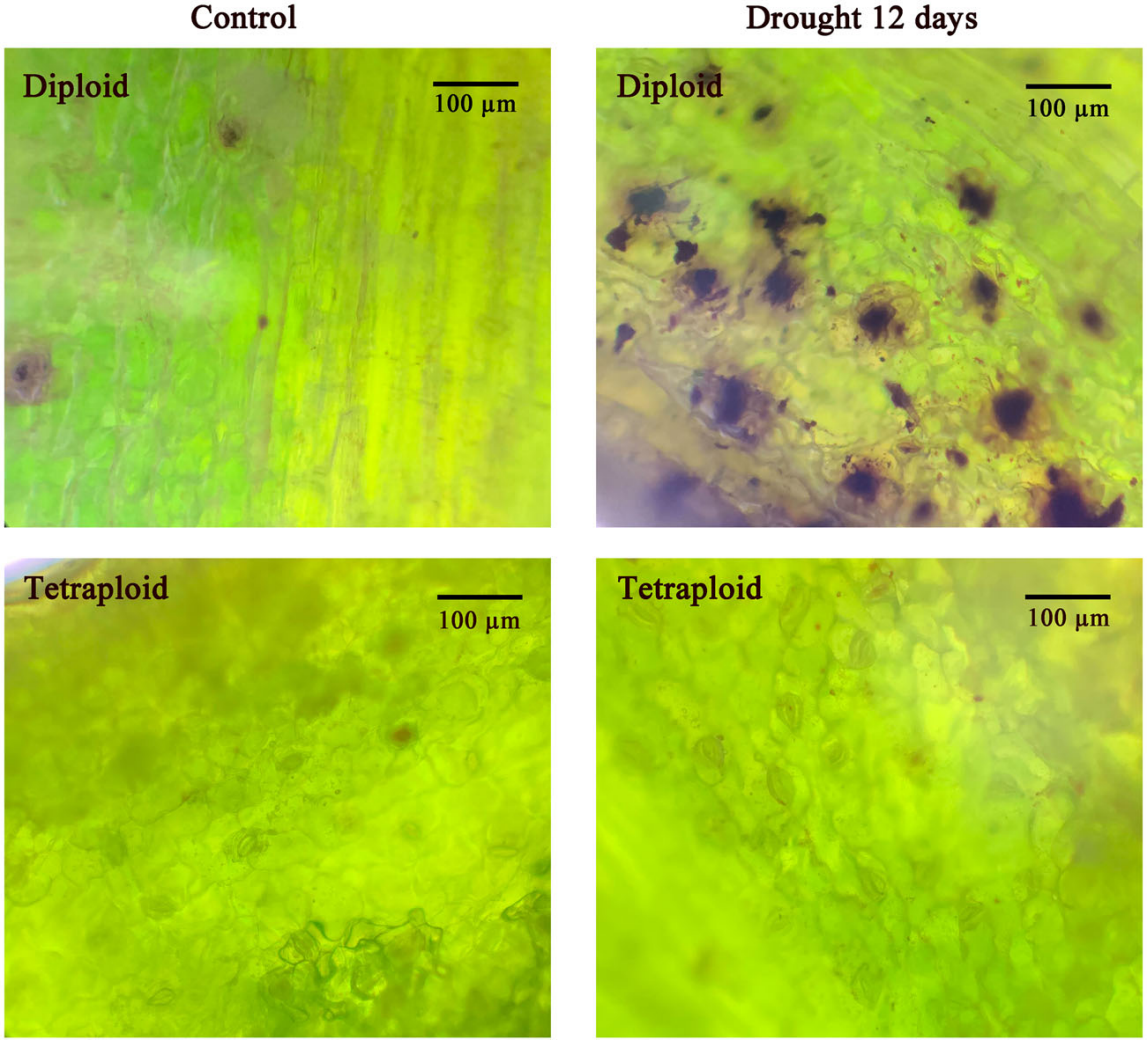
DAB determination in *L. ruthenicum* of different ploidies after 0 days and 12 days of drought. Bars = 100 µm

Chlorophyll and hydrogen peroxide contents can be used as indicators to judge the intensity of resistance to drought stress. Diploid plants showed similar trends in chlorophyll *a* and *b*, total chlorophyll, and carotenoid contents, which began to decline on the 12th day of treatment (Fig. 3). These results indicated that the diploid plants were unable to maintain normal growth and that chlorophyll synthesis was inhibited as a result. The pigment contents of the tetraploid plants showed a continuous increasing trend revealing that on the 12th day, the plants were at an early stage of mild stress, which promoted water absorption by the plants and permitted the continued synthesis of chlorophyll *a* and *b* (Fig. 3). Excessive concentrations of hydrogen peroxide, which is a reactive oxygen species, cause oxidative damage to cells. The microscopic examination of DAB-stained leaves revealed that *L. ruthenicum* plants of each ploidy accumulated only a small amount of hydrogen peroxide under an adequate water supply. After 12 days of drought stress, black precipitates accumulated in the stomata of the diploid plants, but only small amounts of precipitate accumulated in the tetraploid plants. This observation suggests that the oxidative damage caused by drought stress was less severe in the tetraploid plants (Fig. 4).

### Endogenous ABA contents in diploid and tetraploid plants under natural conditions

Abscisic acid is an important phytohormone with multiple functions. ABA is not only critical to plant growth and development but also plays a pivotal role in plant resistance and tolerance to pernicious environmental stresses. The results of UPLC-ESI-MS/MS analysis revealed that polyploidization considerably increased the endogenous ABA content of tetraploids in non-adverse environments (Fig. 2c; Supplementary Fig. S1).

### Overview of SMRT and Illumina sequencing

Using SMRT sequencing technology, 23.04 Gb of subreads were ultimately scanned. After screening according to the criteria of full passes ≥0 and quality >0.90, 640,797 CCS sequences were extracted from the original sequences. After preprocessing by removing redundant reads from the generated data, 22,849 full-length transcripts were obtained (Table 1). The leaves from plants of different ploidies grown in the greenhouse for 1 month were sampled. The cDNA libraries were sequenced independently with three replicates to generate 28.2 Gb of raw data, which yielded 23.7 Gb of high-quality clean data after quality control. The raw data were deposited in the NCBI Sequence Read Archive (SRA) database (PRJNA546099). The nonredundant transcripts generated by the PacBio system were used as a reference for sequence alignment and protein retrieval.

**Table 1:** Full-length transcriptome sequences statistics

	Total number	cDNA size	CCS Number	Number of undesired primer reads	Number of undesired poly-A reads	Number of filtered short reads	Number of full-length non-chimeric reads	Full-length non-chimeric percentage (FLNC%)
SMRT	22849	1–6 K	640797	79425	427635	818	507060	79.13

**Fig. 5:**
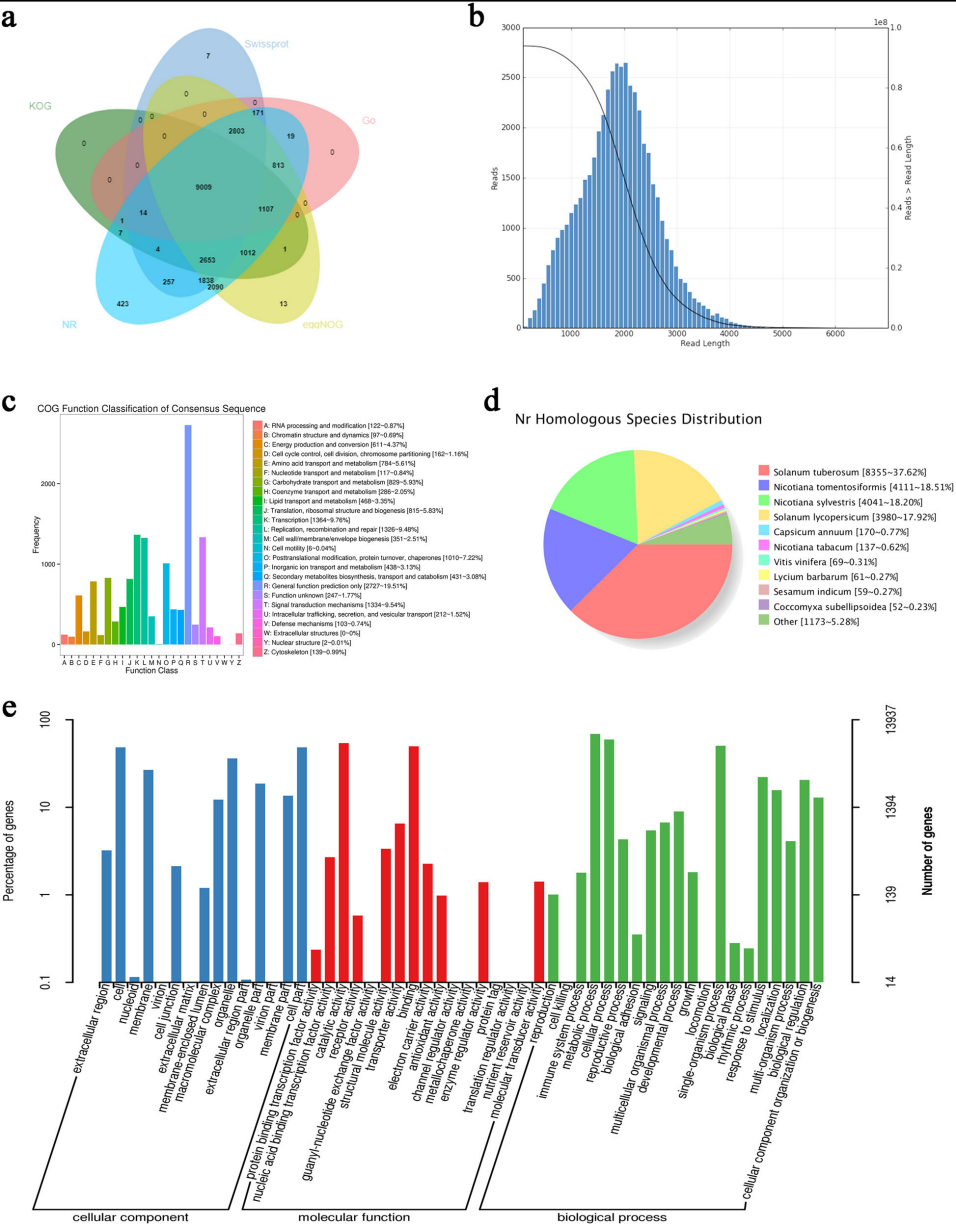
Annotation information for full-length transcripts in multiple databases. **a** Annotation distribution of full-length transcripts in five databases: KOG, SwissProt, Go, NR, and eggNOG. **b** Consensus isoform sequence length distribution of full-length transcripts. **c** Functional classification of consensus isoform sequences in the COG database. **d** Homologous species distribution in the Nr database. **e** Functional classification of consensus isoform sequences in the GO database

To explore the functions of the unigenes and obtain annotation information for the transcripts, a BLAST search was conducted. Functional annotations were performed in multiple public databases, including the National Center for Biotechnology Information Nr, KEGG, GO, COG, Swiss-Prot, KOG, Pfam, and Evolutionary Genealogy of Genes: Nonsupervised Orthologous Groups (eggNOG) databases. A total of 46,997 transcripts were identified in the seven databases: 46,725 in Nr (99.4%), 20,323 in COG (43.5%), 29,308 in GO (62.7%), 20,987 in KEGG (44.9%), 29,242 in KOG (62.6%), 40,910 in Pfam (87.6%), 35,506 in SwissProt (76.0%), and 45,103 in eggNOG (96.5%) (Fig. 5).

### Differential expression of genes in diploid and autotetraploid plants

**Fig. 6:**
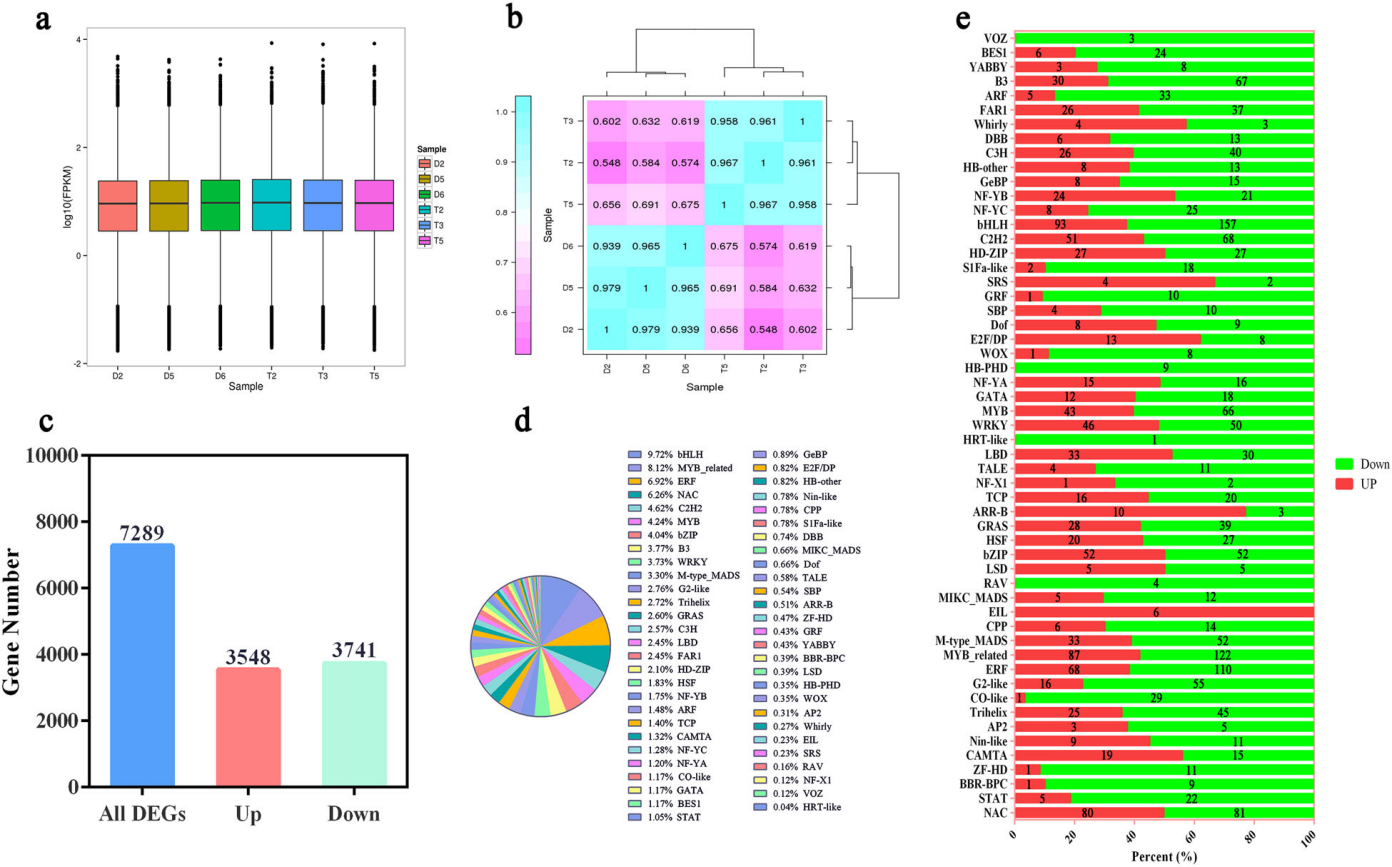
Summary of Illumina sequencing based on the full-length transcriptome. **a** FPKM box plot of the second-generation transcriptome of each sample. **b** Correlation heat map of expression in two pairs of samples according to next-generation sequencing. **c** Summary of the DEGs according to the second-generation transcriptome. **d** Statistics of differentially expressed TFs in *L. ruthenicum* of different ploidies. **e** Investigation of the up- and downregulation of differentially expressed TFs

**Fig. 7:**
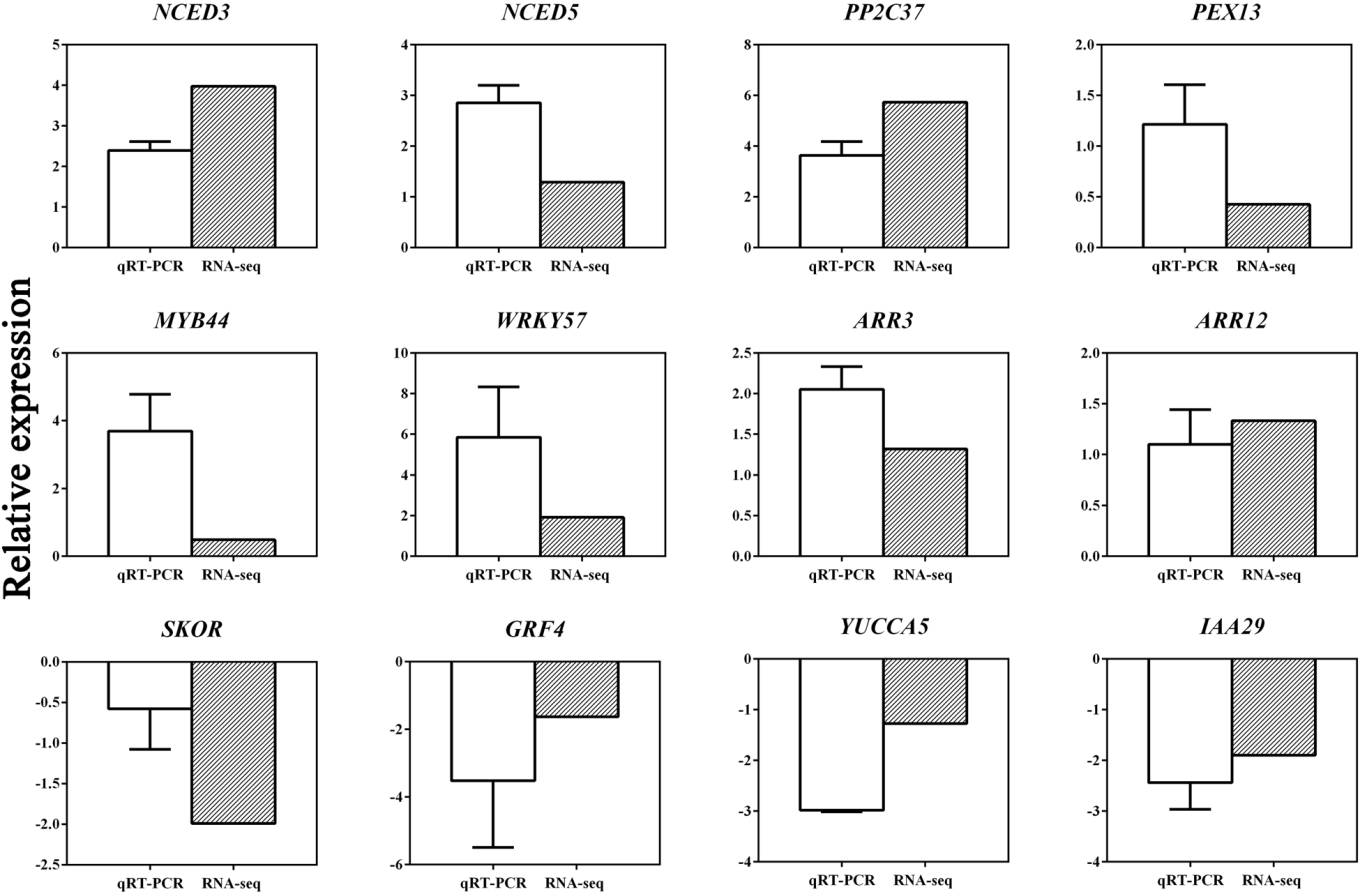
Expression patterns of 12 candidate genes according to RNA-seq (white) and qRT-PCR (oblique line) for selected transcripts. The data represent the mean ± SD of three independent experiments. The *X*-axis shows the selected gene ID, and the *Y*-axis shows the log_2_ ratio

The FPKM distribution of the next-generation transcriptome sequence data was visualized in a box plot to compare the overall transcript expression levels of the different samples. Gene expression between the diploid and autotetraploid plants was stable (Fig. 6a). A correlation heat map was generated to reflect the hierarchical clustering among the samples. The identical samples exhibited excellent repeatability, and dissimilar samples were divided into two clusters (Fig. 6b). Overall, the results confirmed the high accuracy of transcriptome sequencing. In addition to determining the accuracy of the transcriptome data measured through bioinformatics verification, we screened 12 unigenes associated with hormones and stress to quantify their expression levels by qRT-PCR analysis. The internal reference gene used for such analyses is generally a member of the stably expressed *Actin* gene family. *Actin* expression was altered in the tetraploid plants in comparison with that in diploid plants after chromosome doubling. Therefore, *Actin7*, which was not affected by ploidy, was selected as the internal reference gene. The qRT-PCR analysis of the 12 unigenes in the materials of two different ploidy levels was consistent with the RNA-Seq data, which demonstrated the credibility of the transcriptome data (Fig. 7).

We detected 7289 DEGs that showed significant differences in expression between diploid and tetraploid plants, among which 3548 were upregulated, and 3741 were downregulated (Fig. 6c). Among the upregulated DEGs, the top 50 transcripts were classified as TFs, protein kinases, and functional genes according to their types and were further categorized as stress resistance-, growth-, and metabolism-related according to their functional division. The most noteworthy difference was observed for the homeobox-leucine zipper protein *ATHB-12*, which is a component of a pathway specific to ion homeostasis and can be induced by ABA and NaCl (Table S2). TFs such as ERF, NAC, MYB, DREB, and HD-ZIP were associated with the stress response, and a MAPKKK protein kinase exhibited distinctly increased expression under the influence of polyploidization.

### Differentially expressed proteins in diploid and autotetraploid plants

**Fig. 8:**
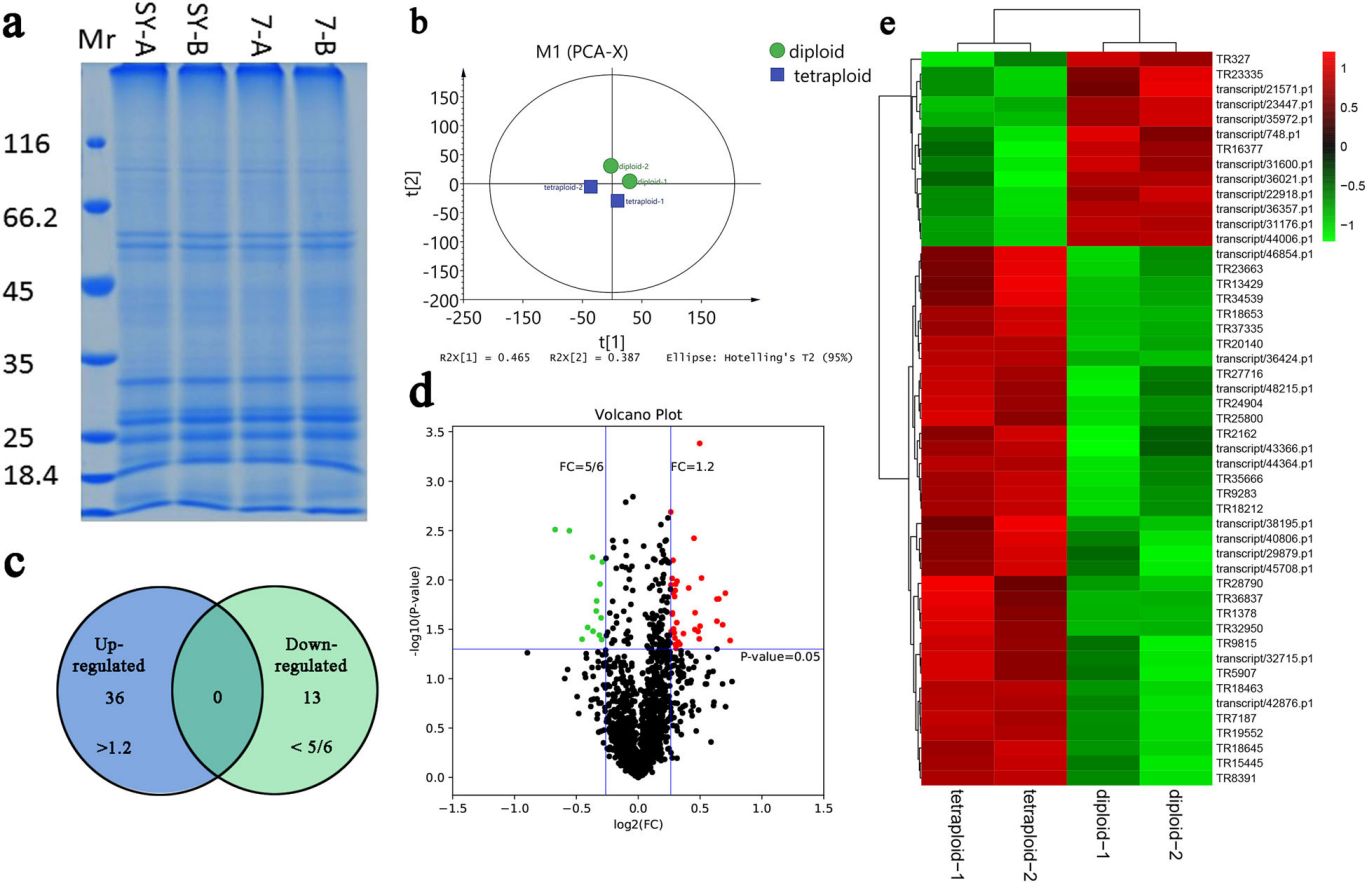
Summary of proteomic results. **a** SDS-PAGE of proteomics samples. **b** PCA of proteomics samples. **c** Venn diagram of differentially expressed proteins according to proteomic analysis. **d** Venn diagram of differentially expressed proteins according to proteomic analysis. **e** Hierarchical clustering of differentially expressed proteins according to proteomic analysis

The molecular weight of the proteins in a sample can be determined by SDS-PAGE via proteomics. The protein bands of the *L. ruthenicum* samples were further separated in gels by molecular weight (Fig. 8a). Subsequently, to verify the data reliability and to screen the credible data for further analysis, we performed a principal component analysis (PCA) as a quality control procedure on the raw data. The results showed that the repetitive bands in diploid and tetraploid plants were grouped in the same cluster, indicating that the experimental data were generally reliable and of high quality (Fig. 8b). The nonredundant transcripts served as a benchmark for protein mining and were ultimately used to retrieve raw data from the proteome to obtain 2716 proteins showing qualitative differences and 2367 proteins showing quantitative differences. In total, 1599 authentic proteins and 49 DEPs were filtered according to the determined screening criteria. Among the DEPs whose differences were triggered by genome doubling, 36 were significantly upregulated and 13 were downregulated, indicating that polyploidization may downregulate abundant transcripts, but at the posttranslational level, most of the DEPs were upregulated (Fig. 8c–e). Based on functional annotations, approximately half of the top 10 DEPs were classified as stress responsive. In particular, a pathogenesis-related (PR) protein, osmotin-like protein, and a heat shock cognate protein were strongly differentially expressed between diploid and tetraploid plants, revealing that the chromosome doubling event regulated the expression of stress-related proteins at the post-transcriptional level (Table 2).

**Table 2:** List of differential expression proteins between diploid and tetraploid

Accession	Fold change	Annotation	Coverage (%)	MW (kDa)	pI
TR23663	1.6738	Glucan endo-1,3-beta-glucosidase, basic vacuolar isoform	19	41.6	7.23
TR35666	1.6298	Pathogenesis-related protein R major form	9	15.8	5.06
TR9815	1.6052	T-complex protein 1 subunit zeta-like	4	61.9	6.44
TR32950	1.5707	Biotic cell death-associated protein	4	22.8	8.51
TR24904	1.5549	Mavicyanin-like	29	13.6	8.46
TR25800	1.5543	Putative S-RNase binding protein p11 precursor	16	12	9.28
TR37335	1.4257	Osmotin-like protein OSML13-like	11	28.2	8.73
Transcript/32715.p1	1.4140	Unknown	20	49.7	4.98
Transcript/36424.p1	1.4111	Uncharacterized protein LOC102600321	9	34	4.86
TR13429	1.4082	Pathogenesis-related protein P2-like precursor	30	17.4	7.62
Transcript/40806.p1	1.3981	Histone H1	9	30.9	10.67
TR1378	1.3753	Mediator of RNA polymerase II transcription subunit 30	28	17.6	8.72
TR36837	1.3739	GDSL esterase/lipase At1g29670-like	15	42.3	6.15
TR18653	1.3669	Histone H4-like	42	13.8	11
TR18645	1.3269	5’-nucleotidase domain-containing protein 4 isoform X2	2	71.1	6.02
Transcript/46854.p1	1.2883	Probable histone H2B.3	42	15.5	10.14
TR28790	1.2630	Oxalate—CoA ligase-like	11	56.5	6.96
Transcript/29879.p1	1.2510	Eukaryotic initiation factor 4A-15-like isoform X2	17	51.6	6.18
TR7187	1.2427	Probable carboxylesterase 120-like	20	36.2	5.83
TR34539	1.2409	60 S ribosomal protein L9-1	37	13	9.55
TR2162	1.2364	F-box protein SKIP23-like	26	25	5.87
TR18212	1.2334	Histone H2A	21	11.7	10.1
Transcript/43366.p1	1.2315	Proteasome subunit beta type-4	11	32.9	9.09
TR8391	1.2303	Protein EXORDIUM-like 2	15	31.6	9.16
Transcript/38195.p1	1.2284	Guanine nucleotide-binding protein subunit beta-like protein	23	37.5	7.9
Transcript/42876.p1	1.2259	Reticulon-like protein B4	13	30.6	8.69
TR5907	1.2241	Ras-related protein Rab11B	23	19	5.66
Transcript/48215.p1	1.2191	Cytochrome b5-like	11	17.7	5.74
Transcript/44364.p1	1.2173	Nectarin-1-like	6	23.2	9.01
TR18463	1.2155	UBP1-associated protein 2C-like	21	47.9	8.68
TR15445	1.2112	Heat shock cognate protein 80	23	80.1	5.05
TR27716	1.2099	Unknown protein DS12 from 2D-PAGE of leaf, chloroplastic	14	31.4	5.16
TR19552	1.2093	Glucose-6-phosphate isomerase 1, chloroplastic-like	8	67.4	5.68
Transcript/45708.p1	1.2026	HMG-Y-related protein B	12	20.3	10.89
TR9283	1.2014	60 S ribosomal protein L9-1-like	46	25.7	9.74
TR20140	1.2002	Endochitinase 3; Flags: Precursor	34	36.6	8.54
Transcript/35972.p1	0.8182	Plastidic aldolase	38	42.9	6.79
Transcript/21571.p1	0.8149	Pectinesterase-like	7	59.6	7.9
Transcript/31600.p1	0.8120	Uncharacterized protein LOC104101491 isoform X1	1	52.3	9.19
Transcript/36357.p1	0.8075	Fructose-1,6-bisphosphatase, cytosolic	26	37.1	5.73
TR16377	0.8042	Phosphoglycolate phosphatase 1B, chloroplastic-like	37	40.9	8.6
Transcript/22918.p1	0.7921	Serine hydroxymethyltransferase, mitochondrial	39	57.2	8.48
TR327	0.7905	Auxin-binding protein ABP19a-like	28	21.6	7.33
Transcript/36021.p1	0.7762	GDP-mannose 3',5'-epimerase	32	44.8	6.95
Transcript/23447.p1	0.7738	Serine hydroxymethyltransferase, mitochondrial	38	61.5	9.07
TR23335	0.7532	11 S globulin subunit beta-like	31	38.8	5.3
Transcript/748.p1	0.7301	J domain-containing protein required for chloroplast accumulation response 1	12	113.6	6.86
Transcript/31176.p1	0.6800	Aminomethyltransferase, mitochondrial	29	44.1	8.56
Transcript/44006.p1	0.6277	Ferritin-3, chloroplastic	8	28.6	5.38

### Prediction of TFs

TFs are important molecules that regulate gene expression; they directly control the extent of gene expression and participate in an extensive range of biological processes. A total of 2573 TFs were detected in this study (Fig. 6d). Different TF families showed significant up- and downregulation. Polyploidization led to changes in the regulatory mechanisms of TFs and thereby enhanced the function of TFs (Fig. 6e). The top differentially expressed TFs were bHLH, ERF, and NAC TFs, which are involved in stress stimulation and tolerance (Fig. 6d). The bHLH TFs are a large family of eukaryotic proteins consisting of six groups distinguished by DNA-binding elements. Abundant bHLHs participate in the positive regulation of the ABA-induced CBF/DREB1 gene family to increase plant stress tolerance. ERF TFs are a subfamily of the AP2/ERF family that is widespread in plants and interacts with *cis*-acting elements in abiotic and biotic stress-responsive genes to participate in various plant responses. The NAC TFs are among the largest families of TFs, which were relatively recently discovered in plants and play an important role in stress responses. In total, 93 bHLH, 68 ERF, and 80 NAC TFs were upregulated in the tetraploid plants.

### Functional annotation of differentially expressed genes and proteins

**Fig. 9:**
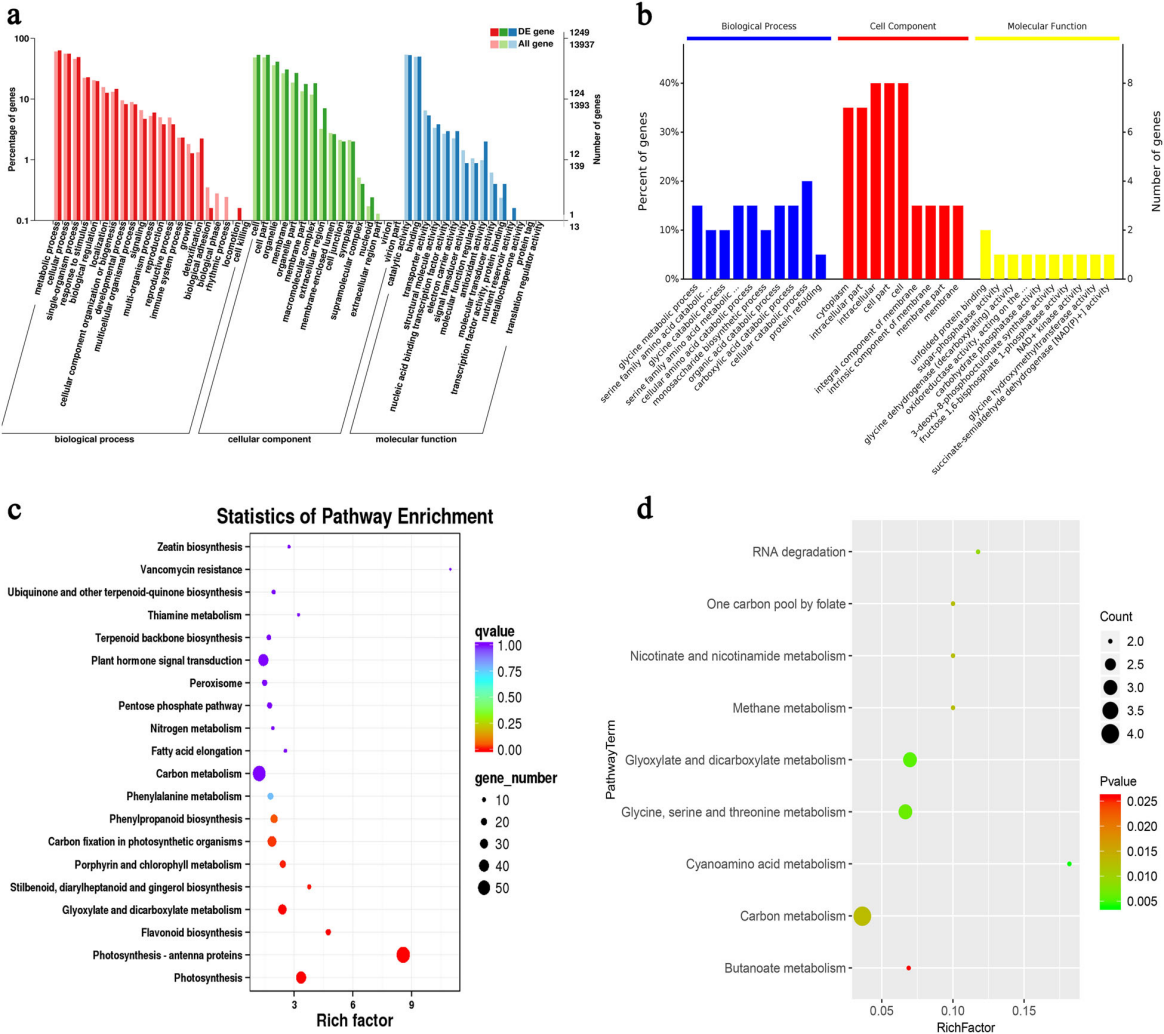
GO and KEGG classification and enrichment of DEGs and DEPs. **a** GO classification of differentially expressed genes. **b** GO classification of differentially expressed proteins. **c** KEGG enrichment of DEGs. **d** KEGG enrichment of DEPs

To determine gene functions that were changed after genome doubling, we conducted GO analysis of DEGs and DEPs between diploid and autotetraploid plants. The GO annotation system consists of three major branches: biological process, molecular function, and cellular component. The GO analysis revealed that the principal biological processes altered by polyploidization corresponded to the “primary and secondary metabolic process” and “response to stimulus” categories, and the main affected molecular function categories were “signal transducer activity” and “antioxidant activity”, regardless of transcriptional or post-transcriptional levels. Biological processes associated with stress resistance were over-represented in the autotetraploid plants, in addition to the enrichment of biosynthetic and metabolic pathways relevant to secondary metabolites. Enriched terms at the gene expression level included “oxidation-reduction process”, “activation of MAPKK activity”, and “abscisic acid-activated signaling pathway”, and those at the protein level included “response to abiotic stimulus” and “primary metabolic process” (Fig. 9a, b). The obstruction of the synthetic pathway for a primary metabolite will affect normal cellular activities.

To analyze whether differentially expressed transcripts and protein profiles were over-represented in a pathway, we performed KEGG enrichment of the significant DEGs and DEPs of the samples with two different ploidy levels. The KEGG classifications and enrichment at the transcript level were mainly focused on plant hormone signal transduction in environmental information processing and the peroxisome in cellular processes, whereas the KEGG enrichment changes in the protein profile were related to the metabolic pathways of primary metabolites (Fig. 9c, d). The multiomics results at the genomic level indicated that changes in the hormone contents of autotetraploids might be an important factor that affects polyploid morphology and stress resistance after chromosome doubling and the regulation of the expression of stress-related proteins at the translational level.

### ABA biosynthesis and signal transduction genes in response to genome doubling

Abscisic acid plays a central role in the environmental adaptability of plants, especially in abiotic stress responses. Under normal growth conditions, a large number of the DEGs identified between tetraploid and diploid plants were associated with ABA, playing roles in processes such as ABA biosynthesis, metabolism, and signal transduction. In the present study, two crucial genes involved in ABA biosynthesis presumably enabled the ABA biosynthesis pathway to be activated and the metabolic pathway to be inhibited; these genes comprised *9-cis-epoxycarotenoid dioxygenase 1* (*NCED1*) and *NCED2*, which were significantly upregulated, and the metabolically vital enzyme *8*’*-hydroxylase*, which was significantly downregulated. The pyrabactin resistance-like 4 receptor (PYL4) in the ABA signal transduction pathway interacts with the C2-domain ABA-related protein (CAR). The significant downregulation of *CAR* led to a similar trend for the ABA receptor. In addition to the induction of the *SNF1-related protein kinase 2* (*SnRK2*) gene, the ABA co-receptor *protein phosphatase 2* *C* (*PP2C*) and the response gene *ABRE-binding factor* (*AREB/ABF*) also showed a negative interaction and exerted undiscovered positive regulatory effects on each other. In addition, ABA is involved in the regulatory mechanism of *ABF*, which rapidly amplifies ABA signaling by significantly inducing the expression of *ABF5-like* genes (Supplementary Table S3). However, the specific implementation of this regulatory mechanism remains unclear.

**Fig. 10:**
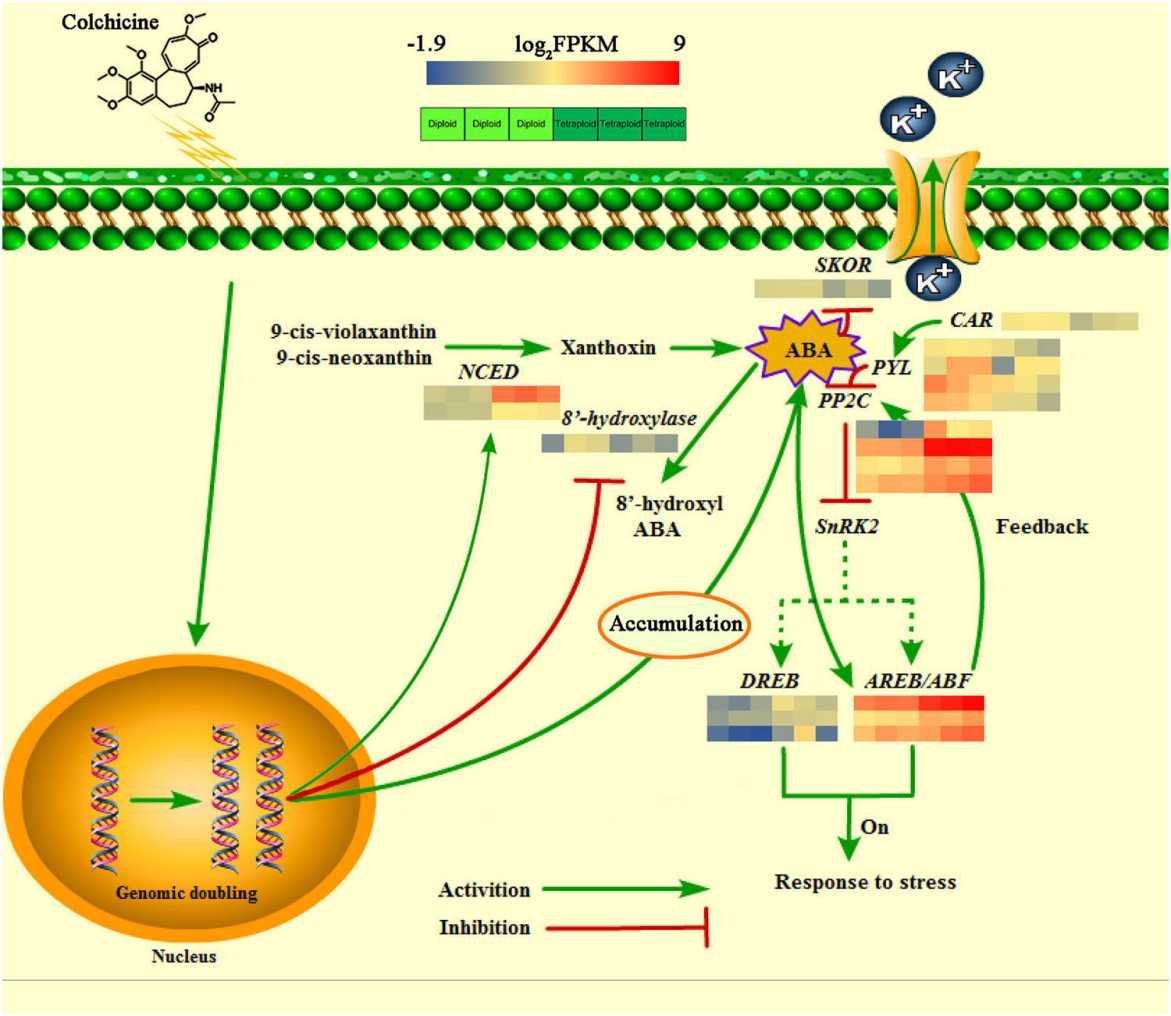
Model for the internal mechanisms of ABA-regulated stress resistance after chromosome doubling. The white boxes contain the sample names (diploid 1, 2, 3 and tetraploid 1, 2, 3)

In general, genome doubling induced ABA accumulation in plant tissues, which may have been due to the changes in the expression of *NCED* and *8’-hydroxylase*, which alter ABA synthesis and metabolic pathways. The continuous accumulation of endogenous ABA activates response genes and stress-resistance genes in the ABA signal transduction pathway to oppose adverse conditions. On the other hand, excessive ABA levels exert a negative feedback effect on ABA signaling to maintain the normal growth and development of the plant (Fig. 10).

### ABA maps to downstream abiotic stress-related DEGs

The accumulation of the stress-related hormone ABA is an output of upstream stress perception and signal transmission and is a key regulator of downstream responses. In turn, ABA can regulate the activity of certain ion channels. The outer rectifier potassium channel *SKOR* is inhibited by ABA to promote the retention of K^+^ in the xylem and maintain the normal water potential of cells. The overexpression of some ABA-responsive proteins, such as late embryogenesis-abundant (LEA) proteins and dehydrin, may be associated with the improved protection of macromolecules and biofilms in tetraploid seedlings. The expression of these proteins was significantly increased in the tetraploid group, presumably to maintain osmotic homeostasis within the cell and preserve normal growth. The transcription factor DREB, as a binding protein for a drought-responsive element, plays an important regulatory role in the molecular responses of plants to drought, high salinity, and low-temperature stresses. The upregulation of *DREB* in tetraploids increases the abundance of DREB, which positively regulates tolerance to abiotic stresses. In addition to ABA-positive regulators, the downregulation of *ICE1* and *HPP1*, which are negatively regulated by ABA-induced signaling, remains critical for the inhibition of growth (Supplementary Table S4).

## Discussion

The generation of polyploids by whole-genome replication triggers major physiological changes relative to the diploid due to greater opportunities for the diversification of gene functions. In addition to organ enlargement, increased stress resistance is a notable alteration induced by polyploidization. Drought is a severe constraint to plant growth and terrestrial ecosystem productivity^40^. Environmental challenges that adversely affect plant growth and productivity will result in diverse physiological responses in plants^41^. In the present study, we not only detected a gradual increase in chlorophyll contents and decreased accumulation of hydrogen peroxide in the tetraploid under drought stress in contrast to the diploid but also demonstrated through phenotypic observations that the autotetraploid exhibits superior resistance to drought. These findings are consistent with previous reports that tetraploids of many plant species show increased resistance to salt^42,43^, drought^8^, and high temperature stresses^44^.

As sessile organisms, plants are subjected to diverse stresses^45^. The flexible coordination of plant growth and development is required to optimize vigor and adaptability in a changing environment through rapid and appropriate responses to stresses^46^. Plants have evolved a sophisticated adaptive system that is mediated by highly complex molecular systems involved in hormonal signaling and metabolism to respond to various adverse conditions, particularly involving the main stress-related hormone ABA and ABA-dependent gene expression^47^. The ABA contents of the autotetraploid and diploid under normal growth conditions were measured because this phytohormone is a pivotal regulator of abiotic stress responses in plants^48^. The ABA content of the tetraploid was 78.4% higher than that of the diploid, suggesting that ABA may be a vital factor in the increased stress resistance of the tetraploid.

The correlation between transcriptomes and proteomes enables the analysis of physiological and biochemical changes to understand plant phenotypes and functions from a molecular perspective. In the present study, we performed a comparative high-throughput omics analysis of diploid and autotetraploid plants under natural conditions using SMRT, Illumina RNA-Seq and iTRAQ technologies. The results revealed molecular differences between diploid and tetraploid plants, verified the association of ABA with increased stress resistance at the transcriptional level and showed that numerous stress-related proteins were strongly affected by genome doubling. Interestingly, we observed that the DEGs identified between the two ploidy levels were markedly enriched in the stress response and hormonal signal transduction GO categories; the majority of the DEGs were closely associated with ABA. These results are similar to the enrichment in proteins responsive to ABA observed in tetraploid citrus^8,49^. The significant increase in ABA content led us to focus on ABA biosynthesis and catabolism. The de novo biosynthesis of ABA involves the use of violaxanthin and neoxanthin as in vivo substrates, which are catalyzed by NCEDs to produce xanthotoxin, the rate-limiting compound^50^. The catabolism of ABA is achieved by ABA hydroxylation mediated by a P450-type monooxygenase, and 8’-hydroxylation is the predominant hydroxylation pathway. Hydroxylated ABA is subsequently converted into a biologically inactive phase acid by spontaneous isomerization^50^. *NCED* and *8’-hydroxylase* act as the rate-limiting gene for ABA biosynthesis and the primary gene for metabolic hydroxylation, respectively, which are strictly controlled by developmental and stress conditions. In the current study, two *NCED* genes (*NCED1* and *NCED2*) were found to be significantly upregulated and one *8’-hydroxylase* gene to be significantly downregulated in tetraploids, which may be a determinant of ABA accumulation in tetraploids at the gene level. The transcriptome results were identical to the experimental results.

In the tetraploid, ABA signaling was substantially altered in response to ABA accumulation. Fifteen genes associated with ABA signaling showed significant changes in expression. The predominant type of ABA receptor in the existing ABA signal transduction model is PYR/PYL/RCARs, which sense ABA intracellularly and form a ternary complex with *PP2C* to regulate downstream *SnRK2*, triggering the subsequent expression of ABA-responsive genes (*AREB/ABF*)^51^. The CAR proteins comprise a small family of lipid-binding C2 domains, which are a novel interaction partner of PYL4 and positively regulate ABA sensitivity^52^. *PYL4* is the major form of ABA receptor and is significantly downregulated in tetraploids. The downregulation of *PYL4* is caused by the downregulation of *CAR* and promotes the subsequent upregulation of *PP2C*, without the activation or inhibition of *SnRK2*. Three *ABF5* and three *DREB* genes were significantly upregulated, indicating that the increase in ABA content induced by genome doubling directly led to the upregulation of ABA-responsive genes and activation of downstream stress-resistance genes. The ABA co-receptor PP2C and the AREB/ABF response genes also show negative interaction and undiscovered positive regulatory effects on each other. To maintain normal growth and development, the activated ABA signal forms a negative feedback loop that enables ABFs to directly bind to the promoter of *PP2C* and mediate the significant upregulation of *PP2C* induced by ABA. An updated model has been proposed in plants highlighting the role of PP2C as an essential co-receptor to increase ABA binding affinity^53^. In the presence of PP2Cs, the binding affinity of PYR/PYL/RCAR to ABA increased 10-fold. Therefore, the upregulated expression of PP2Cs, as coreceptors of ABA, may increase the ability of PYL to bind to ABA, in addition to maintaining growth homeostasis, promoting ABF-induced PP2C repressor expression, and producing negative feedback of ABA signaling. ABA can induce SnRK2 phosphorylation in ABFs, which is an important regulatory mechanism of ABA-activated ABFs. Wang et al.^53^ observed that ABA can dramatically induce the protein accumulation of ABFs, which is achieved via the significant induction of *ABF* gene expression by ABA.

The stress responses of plants are mediated by ABA-dependent and ABA-independent pathways. The transmission of upstream signals can stimulate the responses of downstream genes to counteract adverse conditions. ABA strongly inhibits *SKOR* expression, and the inhibition of *SKOR* can control the transfer of K^+^ to the xylem of the shoots, which is hypothesized to be a mechanism by which plants respond to water stress^2^. DREB is an important TF that induces abiotic stress-related genes and confers stress tolerance in plants. *AtDREB1B* can be induced by exogenous ABA and various stress treatments in *Arabidopsis*^54^. The overexpression of *ABRE* suggests that ABA may play an important role in regulating the expression of DREB TFs as well as the expression of reactive genes in an ABA-independent manner^55^. The *hhp1* mutant shows higher sensitivity to ABA and osmotic stress, as indicated by the germination rate and postemergence growth rate, which demonstrates that *HHP1* is a negative regulator of ABA-dependent signaling^56^. The *ice1* mutant of *Arabidopsis* shows increased induction of ABA signaling, suggesting that *ICE1* is a negative regulator of ABA-dependent responses, in addition to its known role in regulating low-temperature responses, stomatal development, and endosperm decomposition^57^. LEA proteins are associated with abiotic stress resistance in a variety of organisms. These proteins function in the resistance to cell structural collapse and protection of cells from drought and other stresses. The overexpression of *OsLEA5* causes the accumulation of ABA and increases salinity and drought tolerance, whereas the silencing of *OsLEA5* inhibits ABA accumulation and confers reduced stress tolerance; in this regard, *LEA5* is a type of ABA-dependent response gene^58^. The dehydrin gene is a water stress-related gene that belongs to the LEA D-II family and can be induced by exogenous ABA; the encoded protein is also known as the RAB (responsive to ABA) protein^59^. in summary, ABA can induce gene expression in response to multiple stress conditions and increases the advantage of the tetraploid in detrimental environments.

Flavonoid 3’-monooxygenase and cyanidin-3-*O*-glucoside 2-*O*-glucuronosyltransferase were found to be highly expressed; these flavonoid pathway genes are involved in plant growth and secondary metabolite synthesis. Hence, at the transcriptome level, chromosome doubling may influence the accumulation of internal secondary metabolites while increasing stress resistance in tetraploid plants. Secondary metabolic processes are considered to be the outcome of plant ecological and environmental adaptation during long-term evolution and play an important role in balancing the relationship between plants and the ecological environment. Adaptation to the external environment can result in the accumulation of large amounts of secondary metabolites to increase the immunity and resistance of plants.

In addition to transcriptional changes, variations in translation levels are also intimately related to adversity. Two PR proteins that are responsive to pathogen attack and accumulate in the intercellular spaces of many plants are significantly upregulated in tetraploids^60^. The response to water deficits starts with a reduction in cell expansion as a result of the loss of cell turgor. Plants undergo active osmotic adjustment by producing osmolytes to maintain high turgor^61^. Osmotin and osmotin-like proteins, which belong to the PR-5 family, can be regulated by NaCl, ABA, and fungal infection^62^. Transformants overexpressing osmotin and osmotin-like genes exhibit increased salt tolerance in tobacco^63^, tomato^64^, and other Solanaceae species by maintaining chlorophyll contents and preventing the accumulation of reactive oxygen species in comparison with controls.

In conclusion, by monitoring the phenotypic, hormonal, and molecular changes induced by chromosome doubling, we determined that tetraploids exhibit superior drought resistance to diploids and that the internal environmental adaptation of tetraploids differed dramatically from that of diploids under normal growth conditions. Large amounts of ABA accumulate in tetraploids as a result of transcriptional variation in ABA biosynthesis and metabolic pathways and strongly induce the expression of osmotic proteins to increase the drought tolerance of plants at the translational level. The intrinsic mechanisms by which ABA affects the stress resistance of tetraploid and diploid plants were further elucidated to better understand the physiological and molecular mechanisms that increase stress tolerance in polyploid plants. Future emphasis will be placed on the further validation of the proposed polyploid hypothesis model and the investigation of whether the underlying origin of the large differences in ABA-related pathways between diploids and tetraploids represents a chromosomal dosage effect.

## Supplementary information

Genes with regarding primer sequences used for qRT-PCR

Top 50 up-regulated DEGs in tetraploid

Differentially expressed genes associated with ABA biosynthesis, metabolism and signal transduction

Differentially expressed genes associated with ABA biosynthesis, metabolism and signal transduction

Endogenous and standard ABA content determination of different ploidy L. ruthencium

## References

[1] Wu F, Shu J, Jin W (2014). Identification and validation of miRNAs associated with the resistance of maize (*Zea mays* L.) to *Exserohilum turcicum*. PLoS ONE.

[2] Zhu JK (2016). Abiotic stress signaling and responses in plants. Cell.

[3] Sattler MC, Carvalho CR, Clarindo WR (2016). The polyploidy and its key role in plant breeding. Planta.

[4] Song Q, Chen ZJ (2015). Epigenetic and developmental regulation in plant polyploids. Curr. Opin. Plant Biol..

[5] Xu J, Jin J, Zhao H, Li K (2018). Drought stress tolerance analysis of *Populus ussuriensis* clones with different ploidies. J. Forestry Res..

[6] Xu C (2016). In vitro tetraploid plants regeneration from leaf explants of multiple genotypes in populus. Plant Cell Tissue Organ Cult..

[7] Meng F (2016). Physiological and proteomic responses to salt stress in chloroplasts of diploid and tetraploid black locust (*Robinia pseudoacacia* L.). Sci. Rep..

[8] Allario T (2011). Large changes in anatomy and physiology between diploid Rangpur lime (*Citrus limonia*) and its autotetraploid are not associated with large changes in leaf gene expression. J. Exp. Bot..

[9] Chen JH (2017). Physiological characterization, transcriptomic profiling, and microsatellite marker mining of *Lycium ruthenicum*. J. Zhejiang Universityence B.

[10] Liu Y (2014). Comparative analysis of carotenoid accumulation in two goji (*Lycium barbarum* L. and *L. ruthenicum* Murr.) fruits. BMC Plant Biol..

[11] Guo YY (2016). Effects of drought stress on growth and chlorophyll fluorescence of *Lycium ruthenicum* Murr. seedlings. Photosynthetica.

[12] Zeng S (2014). Comparative analysis of anthocyanin biosynthesis during fruit development in two Lycium species. Physiol. Plant.

[13] Prathi NB (2018). Proteomic and transcriptomic approaches to identify resistance and susceptibility related proteins in contrasting rice genotypes infected with fungal pathogen *Rhizoctonia solani*. Plant Physiol. Biochem.

[14] Zhang J (2018). A full-length transcriptome of *Sepia esculenta* using a combination of single-molecule long-read (SMRT) and Illumina sequencing. Mar. Genomics.

[15] Luo D (2019). Full-length transcript sequencing and comparative transcriptomic analysis to evaluate the contribution of osmotic and ionic stress components towards salinity tolerance in the roots of cultivated alfalfa (*Medicago sativa* L.). BMC Plant Biol..

[16] Gao S (2017). Two novel lncRNAs discovered in human mitochondrial DNA using PacBio full-length transcriptome data. Mitochondrion.

[17] Zhao Q (2018). Transcriptome comparative analysis of salt stress responsiveness in chrysanthemum (*Dendranthema grandiflorum*) roots by Illumina- and single-molecule real-time-based RNA sequencing. DNA Cell Biol..

[18] Zhu C, Li X, Zheng J (2018). Transcriptome profiling using Illumina- and SMRT-based RNA-seq of hot pepper for in-depth understanding of genes involved in CMV infection. Gene.

[19] Lou H (2019). Full-length transcriptome analysis of the genes involved in tocopherol biosynthesis in *Torreya grandis*. J. Agric Food Chem..

[20] Mahadevan C (2016). Transcriptome-assisted label-free quantitative proteomics analysis reveals novel insights into piper nigrum-*Phytophthora capsici* phytopathosystem. Front. Plant Sci..

[21] Rao S, Kang X, Li J, Chen J (2019). Induction, identification and characterization of tetraploidy in *Lycium ruthenicum*. Breed. Sci..

[22] Hsiao TC (1973). Plant response to water stress. Annu. Rev. Plant Physiol..

[23] Xiang Y (2015). An ultrahigh-performance liquid chromatography method with electrospray ionization tandem mass spectrometry for simultaneous quantification of five phytohormones in medicinal plant *Glycyrrhiza uralensis* under abscisic acid stress. J. Nat. Med..

[24] Li W (2006). Fast program for clustering and comparing Large Sets of Protein or Nucleotide Sequences. Bioinformatics.

[25] Deng Y (2006). Integrated nr database in protein annotation system and its localization. Computer Eng..

[26] Apweiler R (2004). UniProt: the universal protein knowledgebase. Nucleic Acids Res..

[27] Ashburner M (2000). Gene ontology: tool for the unification of biology. Nat. Genet..

[28] Tatusov RL, Galperin MY, Natale DA, Koonin EV (2000). The COG database: a tool for genome-scale analysis of protein functions and evolution. Nucleic Acids Res..

[29] Koonin EV (2004). A comprehensive evolutionary classification of proteins encoded in complete eukaryotic genomes. Genome Biol..

[30] Finn RD (2014). Pfam: the protein families database. Nucleic Acids Res..

[31] Kanehisa M (2004). The KEGG resource for deciphering the genome. Nucleic Acids Res..

[32] Altschul SF (1997). Gapped BLAST and PSI-BLAST: a new generation of protein database search programs. Nucleic Acids Res..

[33] Martin M (2011). Cutadapt removes adapter sequences from high-throughput sequencing reads. Embnet J..

[34] Li B, Dewey CN (2011). RSEM: accurate transcript quantification from RNA-Seq data with or without a reference genome. BMC Bioinforma..

[35] Smith PK, Krohn RI, Hermanson GT (1985). Measurement of protein using bicinchoninic acid. Anal. Biochem..

[36] Candiano G (2004). Blue silver: a very sensitive colloidal Coomassie G-250 staining for proteome analysis. Electrophoresis.

[37] Anders S, Huber W (2010). Differential expression analysis for sequence count data. Genome Biol..

[38] Zheng Y (2016). iTAK: A program for genome-wide prediction and classification of plant transcription factors,transcriptional regulators, and protein kinases. Mol. Plant.

[39] Livak KJ, Schmittgen TD (2001). Analysis of relative gene expression data using real-time quantitative PCR and the 2(-Delta Delta C(T)) Method. Methods.

[40] Wang X, Komatsu S (2018). Proteomic approaches to uncover the flooding and drought stress response mechanisms in soybean. J. Proteom..

[41] Zandalinas SI (2018). Plant adaptations to the combination of drought and high temperatures. Physiol. Plant.

[42] Fan G (2016). Comparative analysis and identification of miRNAs and their target genes responsive to salt stress in diploid and tetraploid *Paulownia fortunei* seedlings. PLoS ONE.

[43] Liu B, Sun G (2017). MicroRNAs contribute to enhanced salt adaptation of the autopolyploid *Hordeum bulbosum* compared to its diploid ancestor. Plant J..

[44] Zhang XY, Hu CG, Yao JL (2010). Tetraploidization of diploid dioscorea results in activation of the antioxidant defense system and increased heat tolerance. J. Plant Physiol..

[45] Ku YS, Sintaha M, Cheung MY, Lam HM (2018). Plant hormone signaling crosstalks between biotic and abiotic stress responses. Int. J. Mol. Sci..

[46] Xia XJ (2015). Interplay between reactive oxygen species and hormones in the control of plant development and stress tolerance. J. Exp. Bot..

[47] Urano K (2017). Analysis of plant hormone profiles in response to moderate dehydration stress. Plant J..

[48] Zhou L (2014). Exogenous abscisic acid significantly affects proteome in tea plant (*Camellia sinensis*) exposed to drought stress. Hortic. Res..

[49] Wei T (2018). Enhanced ROS scavenging and sugar accumulation contribute to drought tolerance of naturally occurring autotetraploids in *Poncirus trifoliata*. Plant Biotechnol. J..

[50] Ting D, Youngmin P, Inhwan H (2015). Abscisic acid: biosynthesis, inactivation, homoeostasis and signalling. Essays Biochem..

[51] Lee SC, Luan S (2011). ABA signal transduction at the crossroad of biotic and abiotic stress responses. Plant Cell Environ..

[52] Lesia R (2014). C2-domain abscisic acid-related proteins mediate the interaction of PYR/PYL/RCAR abscisic acid receptors with the plasma membrane and regulate abscisic acid sensitivity in Arabidopsis. Plant Cell.

[53] Wang X (2019). ABRE-BINDING FACTORS play a role in the feedback regulation of ABA signaling by mediating rapid ABA induction of ABA co-receptor genes. N. Phytol..

[54] Wei T (2016). Arabidopsis DREB1B in transgenic *Salvia miltiorrhiza* increased tolerance to drought stress without stunting growth. Plant Physiol. Biochem..

[55] Amrita S, Sameet M, Angelica L, Sujata B (2010). Over-represented promoter motifs in abiotic stress-induced *DREB* genes of rice and sorghum and their probable role in regulation of gene expression. Plant Signal Behav..

[56] Chen CC, Liang CS, Kao AL, Yang CC (2009). HHP1 is involved in osmotic stress sensitivity in Arabidopsis. J. Exp. Bot..

[57] Liang CH, Yang CC (2015). Identification of ICE1 as a negative regulator of ABA-dependent pathways in seeds and seedlings of Arabidopsis. Plant Mol. Biol..

[58] Huang L (2018). An atypical late embryogenesis abundant protein OsLEA5 plays a positive role in ABA-induced antioxidant defense in *Oryza sativa* L. Plant Cell Physiol..

[59] Bao F (2017). Overexpression of *Prunus mume* dehydrin genes in tobacco enhances tolerance to cold and drought. Front. Plant Sci..

[60] Payne G (1988). Isolation and nucleotide sequence of a novel cDNA clone encoding the major form of pathogenesis-related protein R. Plant Mol. Biol..

[61] Fox H (2018). Transcriptome analysis of *Pinus halepensis* under drought stress and during recovery. Tree Physiol..

[62] Zhu B, Chen TH, Li PH (1995). Activation of two osmotin-like protein genes by abiotic stimuli and fungal pathogen in transgenic potato plants. Plant Physiol..

[63] Sokhansanj A, Noori SAS, Niknam V (2006). Comparison of bacterial and plant genes participating in proline biosynthesis with osmotin gene, with respect to enhancing salinity tolerance of transgenic tobacco plants. Russ. J. Plant Physl.

[64] Goel D (2010). Overexpression of osmotin gene confers tolerance to salt and drought stresses in transgenic tomato (*Solanum lycopersicum* L.). Protoplasma.

