# Supplementary material for: Chromosome doubling mediates superior drought tolerance in Lycium ruthenicum via abscisic acid signaling
Source: Hortic Res. 2020 Apr 1;7:40. doi: 10.1038/s41438-020-0260-1 (PMC7109118; doi:10.1038/s41438-020-0260-1)
Supplement: Supplementary file 1 — Genes with regarding primer sequences used for qRT-PCR [file 41438_2020_260_MOESM1_ESM.docx]

| Table S1. Genes with regarding primer sequences used for qPCR. | | | |
| --- | --- | --- | --- |
| **Unigene** | **Gene Description** | **Forward Primer sequences (5'-3′)** | **Reverse Primer sequences (5'-3′)** |
| TR16793\|c0_g1 | Actin-7 | CTGACGGTGAGGACATTCA | GAGCATCATCTCCAGCAAAG |
| TR7652\|c0_g1 | NCED3 | CACGAACTTGAACACCCTTTAC | CGGGAGTGATTGAGTGACTG |
| TR17678\|c0_g1 | NCED5 | CCCGTTTCGTAGATTCTCC | GACACCACCAGATTCCATT |
| TR29161\|c0_g1 | PP2C37 | TGAGCCAGAGGTGACGATAA | CTTCCCTTTCAGGCACATC |
| TR24610\|c0_g1 | PEX13 | AACCCTCAGGTAGTAGCCCA | TGAAACAATCTCACCAGGC |
| TR5399\|c0_g1 | MYB44 | TTGAGGAAGACGGAGTGTT | AAAGGCTTGCGTGTAAGTG |
| TR4356\|c0_g1 | WRKY57 | CGATGAGGATTTGGTGAGA | GCCGCCGTTATTTCAGTA |
| TR21687\|c0_g2 | ARR3 | CCATCCACTTCCTCTCCATC | TCACAAATCTTGGCTGCTG |
| TR14987\|c0_g1 | ARR12 | TGGACGGGTTTGGAGATT | CGCTTAGACCAGCAGCACTA |
| TR1741\|c0_g1 | SKOR | AGTGTTGTCCTGTGGTTACCT | AAGAGCCTGATGGGAAATC |
| TR21501\|c0_g1 | GRF4 | GGGATTGGGTATGACTATGG | TCCAGCGGAACAGGTAAT |
| TR21819\|c0_g1 | YUCCA5 | CCCTCTCCTTGTGAAACCA | ATGTCCCTTATTGGCTACAGG |
| TR9471\|c0_g1 | IAA29 | TGGAAGGAGTAGCCATAGGAA | AACAGTGTAAAGCCCGCAC |
