# Supplementary material for: Chromosome doubling mediates superior drought tolerance in Lycium ruthenicum via abscisic acid signaling
Source: Hortic Res. 2020 Apr 1;7:40. doi: 10.1038/s41438-020-0260-1 (PMC7109118; doi:10.1038/s41438-020-0260-1)
Supplement: Supplementary file 2 — Top 50 up-regulated DEGs in tetraploid [file 41438_2020_260_MOESM2_ESM.docx]

Table S2. Top 50 up-regulated DEGs in tetraploid.

| Gene ID | Log_2_ Fold Change | Description |
| --- | --- | --- |
|  |  |  |
| TR8398\|c1_g1 | 5.37911 | homeobox-leucine zipper protein ATHB-12-like |
| TR22173\|c0_g1 | 5.30499 | EID1-like F-box protein 3 |
| TR19780\|c0_g1 | 5.26701 | ethylene-responsive transcription factor ERF109-like |
| TR6961\|c0_g2 | 5.25822 | U-box domain-containing protein 19-like |
| TR23627\|c0_g1 | 4.89401 | NAC domain-containing protein 72-like |
| TR28382\|c0_g2 | 4.87452 | transcription factor MYB21-like |
| TR28227\|c0_g1 | 4.85039 | zinc finger protein ZAT12-like |
| TR14440\|c1_g1 | 4.84514 | vicilin-like antimicrobial peptides 2-3 |
| TR13724\|c0_g1 | 4.79805 | homeobox-leucine zipper protein ATHB-12-like |
| TR29161\|c0_g1 | 4.77325 | probable protein phosphatase 2C 24 isoform X1 |
| TR4277\|c0_g1 | 4.6142 | vicilin-like antimicrobial peptides 2-3 |
| TR11781\|c0_g1 | 4.5996 | dehydration-responsive element-binding protein 1E-like |
| TR6871\|c0_g1 | 4.30202 | putative calcium-binding protein CML19 |
| TR946\|c0_g2 | 4.22815 | probable linoleate 9S-lipoxygenase 5 |
| TR22674\|c0_g1 | 4.16486 | probable carboxylesterase 17 |
| TR1239\|c0_g1 | 4.1246 | expansin-like B1-like |
| TR13316\|c0_g1 | 4.09658 | 9-lipoxygenase |
| TR4762\|c0_g2 | 4.07826 | basic leucine zipper 63-like |
| TR29161\|c0_g5 | 4.01158 | probable protein phosphatase 2C 24 |
| TR23059\|c0_g4 | 4.00924 | probable calcium-binding protein CML41-like |
| TR7652\|c0_g1 | 4.00034 | 9-cis-epoxycarotenoid dioxygenase 1 |
| TR2789\|c0_g1 | 3.9218 | protein LURP-one-related 14-like |
| TR6871\|c0_g2 | 3.89652 | putative calcium-binding protein CML19 |
| TR22787\|c0_g8 | 3.83792 | HD-ZIP protein |
| TR37759\|c0_g1 | 3.78257 | ferric reduction oxidase 4-like |
| TR24967\|c0_g1 | 3.68222 | monothiol glutaredoxin-S1-like |
| TR16110\|c0_g1 | 3.66814 | zinc finger BED domain-containing protein DAYSLEEPER-like |
| TR2626\|c0_g2 | 3.5672 | probable linoleate 9S-lipoxygenase 5 |
| TR11909\|c0_g1 | 3.53482 | myosin heavy chain kinase B-like |
| TR12542\|c0_g1 | 3.50528 | jasmonic acid 2 |
| TR1102\|c0_g1 | 3.42609 | probable protein phosphatase 2C 51 |
| TR11049\|c0_g1 | 3.33369 | late embryogenesis abundant protein D-29-like |
| TR22043\|c0_g1 | 3.30941 | expansin-like B1-like |
| TR19\|c0_g1 | 3.27211 | LOB domain-containing protein 41-like |
| TR20203\|c0_g4 | 3.27128 | mitogen-activated protein kinase kinase kinase A-like |
| TR22138\|c0_g1 | 3.25072 | glutathione S-transferase U8-like |
| TR33368\|c0_g1 | 3.24741 | G-type lectin S-receptor-like serine/threonine-protein kinase At5g24080 isoform X1 |
| TR28029\|c0_g1 | 3.22514 | lysosomal amino acid transporter 1 homolog isoform X1 |
| TR15640\|c2_g4 | 3.2144 | profilin-2 |
| TR11868\|c0_g1 | 3.17474 | delta-1-pyrroline-5-carboxylate synthetase |
| TR37564\|c0_g1 | 3.15519 | cyanidin-3-O-glucoside 2-O-glucuronosyltransferase-like |
| TR29235\|c2_g2 | 3.15413 | leucine-rich repeat receptor protein kinase EXS |
| TR11353\|c0_g1 | 3.13396 | probable calcium-binding protein CML44 |
| TR34220\|c0_g1 | 3.10009 | zinc finger protein ZAT10-like |
| TR20646\|c0_g2 | 3.09678 | putative calcium-binding protein CML19 |
| TR11523\|c0_g1 | 3.07907 | flavonoid 3'-monooxygenase-like |
| TR30957\|c0_g1 | 3.07178 | WAT1-related protein At5g07050-like isoform X1 |
| TR26192\|c0_g1 | 3.05214 | auxin-induced protein PCNT115-like |
| TR2644\|c0_g1 | 3.05085 | protein phosphatase 2C 37-like |
