# Supplementary material for: Chromosome doubling mediates superior drought tolerance in Lycium ruthenicum via abscisic acid signaling
Source: Hortic Res. 2020 Apr 1;7:40. doi: 10.1038/s41438-020-0260-1 (PMC7109118; doi:10.1038/s41438-020-0260-1)
Supplement: Supplementary file 3 — Differentially expressed genes associated with ABA biosynthesis, metabolism and signal transduction [file 41438_2020_260_MOESM3_ESM.docx]

Table S3. Differentially expressed genes associated with ABA biosynthesis, metabolism and signal transduction

|  | Gene ID | Log_2_ Fold Change | Description | |
| --- | --- | --- | --- | --- |
| Biosynthesis | NCED | | | |
|  | TR7652\|c0_g1 | 4.00034 | 9-cis-epoxycarotenoid dioxygenase 1 | |
|  | TR17678\|c0_g1 | 1.57879 | 9-cis-epoxy-carotenoid dioxygenase 2 | |
| Metabolism | 8'-hydroxylase | | | |
|  | TR17390\|c0_g1 | -1.47762 | abscisic acid 8'-hydroxylase 2 |  |
| Transduction | PYL | | | |
|  | TR7846\|c0_g1 | -1.22564 | abscisic acid receptor PYL4-like |  |
|  | TR36337\|c0_g1 | -1.42035 | abscisic acid receptor PYL1 |  |
|  | TR13267\|c0_g1 | -1.69975 | abscisic acid receptor PYL4-like |  |
|  | TR13704\|c0_g1 | -1.98087 | abscisic acid receptor PYL4-like |  |
|  | PP2C | | | |
|  | TR29161\|c0_g1 | 4.77325 | probable protein phosphatase 2C 24 isoform X1 |  |
|  | TR2644\|c0_g1 | 3.05085 | protein phosphatase 2C 37-like |  |
|  | TR7767\|c0_g1 | 1.36206 | probable protein phosphatase 2C 50 isoform X1 |  |
|  | TR27544\|c0_g1 | 1.30637 | protein phosphatase 2C 16-like isoform X1 |  |
|  | CAR | | | |
|  | TR21659\|c0_g1 | -1.22464 | Protein C2-DOMAIN ABA-RELATED 3 |  |
|  |  | | | |
|  | TR30017\|c0_g1 | 1.89001 | ABSCISIC ACID-INSENSITIVE 5-like protein 5 |  |
|  | TR26038\|c1_g1 | 1.06824 | ABSCISIC ACID-INSENSITIVE 5-like |  |
|  | TR9105\|c1_g1 | 1.02282 | abscisic acid-insensitive 5-like protein |  |
