# Supplementary material for: Chromosome doubling mediates superior drought tolerance in Lycium ruthenicum via abscisic acid signaling
Source: Hortic Res. 2020 Apr 1;7:40. doi: 10.1038/s41438-020-0260-1 (PMC7109118; doi:10.1038/s41438-020-0260-1)
Supplement: Supplementary file 4 — Differentially expressed genes associated with ABA biosynthesis, metabolism and signal transduction [file 41438_2020_260_MOESM4_ESM.docx]

Table S4. Differentially expressed genes in response to stress resistance in the downstream of ABA

|  | Gene ID | Log_2_ Fold Change | Description |
| --- | --- | --- | --- |
| DREB | TR11781\|c0_g1 | 4.5996 | dehydration-responsive element-binding protein 1E-like |
|  | TR2172\|c1_g1 | 2.20895 | dehydration-responsive element-binding protein 2A-like |
|  | TR17161\|c0_g1 | 1.31659 | dehydration-responsive element-binding protein 2C-like |
| SKOR | TR1741\|c0_g1 | -1.30469 | potassium channel SKOR-like |
| ICE1 | TR20708\|c0_g1 | -1.19496 | transcription factor ICE1-like |
| HHP1 | TR16951\|c0_g1 | -1.62022 | heptahelical transmembrane protein 1-like |
|  | TR38873\|c0_g1 | -2.09551 | heptahelical transmembrane protein 1-like |
| DHN | TR24722\|c0_g1 | 1.41872 | dehydrin DHN1-like |
| LEA | TR11049\|c0_g1 | 3.33369 | late embryogenesis abundant protein D-29-like |
|  | TR2150\|c0_g1 | 2.15505 | late embryogenesis abundant protein Lea5-like |
