# Supplementary figures and images for: Chromosome doubling mediates superior drought tolerance in Lycium ruthenicum via abscisic acid signaling
Source: Hortic Res. 2020 Apr 1;7:40. doi: 10.1038/s41438-020-0260-1 (PMC7109118; doi:10.1038/s41438-020-0260-1)

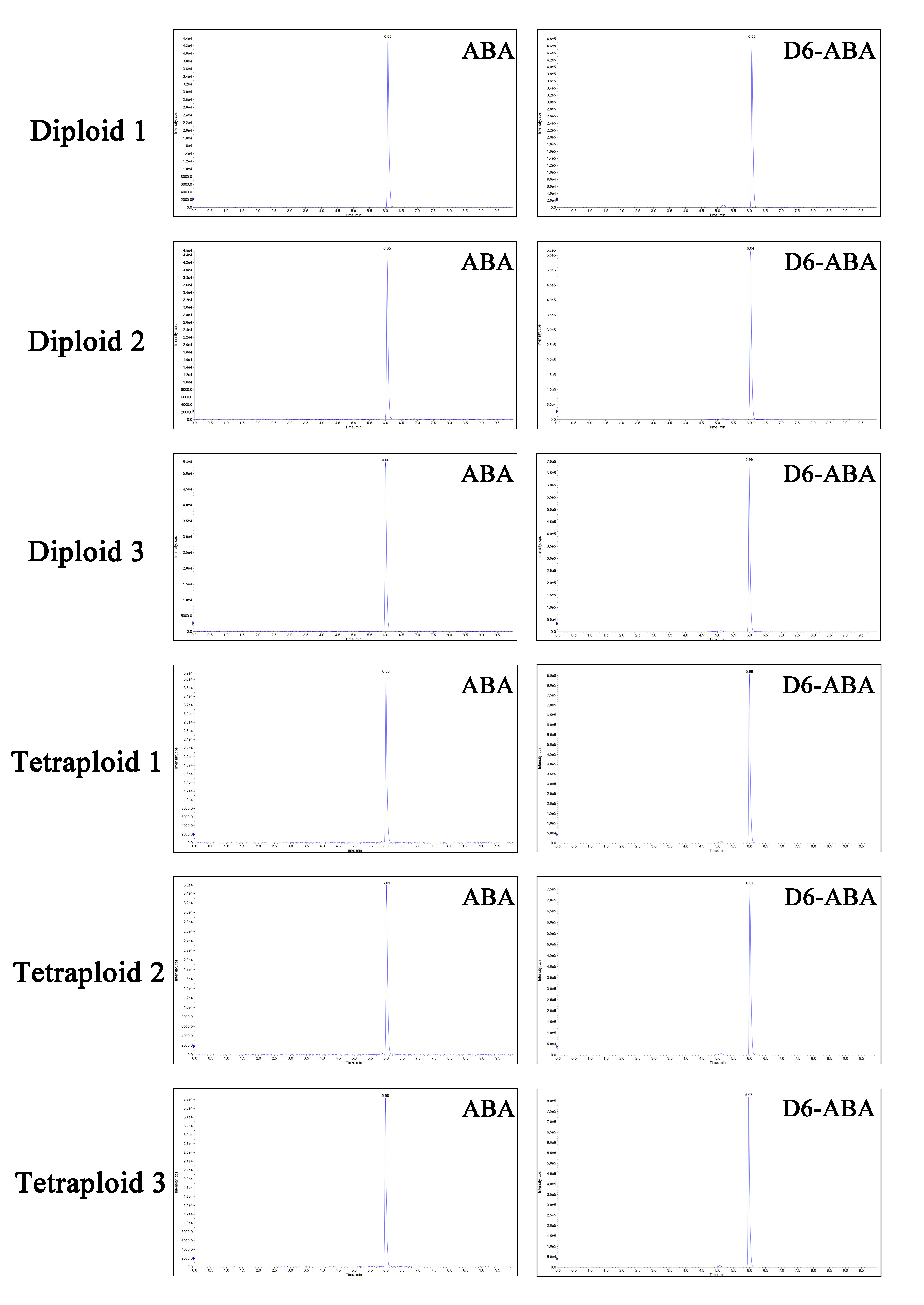

Supplement: Supplementary file 5 — Endogenous and standard ABA content determination of different ploidy L. ruthencium [file 41438_2020_260_MOESM5_ESM.jpg]
